# Supplementary material for: Associations of time of day with cardiovascular disease risk factors measured in older men: results from the British Regional Heart Study
Source: BMJ Open. 2017 Nov 12;7(11):e018264. doi: 10.1136/bmjopen-2017-018264 (PMC5695475; doi:10.1136/bmjopen-2017-018264)
Supplement: Author's manuscript [file bmjopen-2017-018264.draft_revisions.pdf]

BMJ Open

BMJ Open is committed to open peer review. As part of this commitment we make the peer review history of every article we publish publicly available.

When an article is published we post the peer reviewers' comments and the authors' responses online. We also post the versions of the paper that were used during peer review. These are the versions that the peer review comments apply to.

The versions of the paper that follow are the versions that were submitted during the peer review process. They are not the versions of record or the final published versions. They should not be cited or distributed as the published version of this manuscript.

BMJ Open is an open access journal and the full, final, typeset and author-corrected version of record of the manuscript is available on our site with no access controls, subscription charges or pay-per-view fees (http://bmjopen.bmj.com).

If you have any questions on BMJ Open's open peer review process please email editorial.bmjopen@bmj.com

Time of day variations in novel cardiovascular disease risk factors measured in older men

Journal:	*BMJ Open*
Manuscript ID	bmjopen-2017-018264
Article Type:	Research
Date Submitted by the Author:	16-Jun-2017

Complete List of Authors:	Sartini, Claudio; University College of London, Primary Care and Population Health<br>Whincup, Peter; St Georges, University of London, Population Health Research Institute<br>Wannamethee, Goya; University College London<br>Jefferis, Barbara ; University College London<br>Lennon, Lucy; UCL, Department of Primary Care and Population Health<br>Lowe, Gordon; University of Glasgow, Institute of Cardiovascular and Medical Sciences<br>Welsh, Paul; University of Glasgow, Institute of Cardiovascular and Medical Sciences<br>Sattar, Naveed; University of Glasgow, BHF centre<br>Morris, Richard; University of Bristol, School of Social & Community Medicine

**Primary Subject Heading**:	Epidemiology
Secondary Subject Heading:	Public health
Keywords:	biological markers, older adults, cardiovascular disease, diurnal variations

SCHOLARONE™
Manuscripts

Title: Time of day variations in novel cardiovascular disease risk factors measured in older men

Claudio Sartini, MSc [1 *], Peter H Whincup, PhD [2], S Goya Wannamethee, PhD [1], Barbara J Jefferis, PhD [1], Lucy Lennon, MSc [1], Gordon DO Lowe, PhD [3], Paul Welsh, PhD [3], Naveed Sattar, PhD [3], and Richard W Morris, PhD [4]

[1] Department of Primary Care & Population Health, University College London, Rowland Hill Street, NW3 2PF, London. UK

[2] Population Health Research Institute, St George's University of London, Cranmer Terrace London SW17 0RE, UK.

[3] Institute of Cardiovascular and Medical Sciences, University of Glasgow, Glasgow G12 8TA UK

[4] School of Social & Community Medicine, University of Bristol, Canynge Hall, 39 Whatley Rd, Bristol BS8 2PS, UK.

[*] Corresponding Author

E-mail: c.sartini@ucl.ac.uk;

Manuscript number of words: 3897 words (including title page, abstract, manuscript, tables).

For peer review only

Abstract (250 words, now 250)

Objective: We estimated associations of time of day with novel cardiovascular disease (CVD) risk factors measured in older men.

Methods: Novel CVD risk factors (markers of inflammation and haemostasis, and cardiac markers) were measured on one occasion between 08:00-19:00 hours in 4252 men aged 60-79 years from the British Regional Heart Study. Linear models were used to estimate associations between time of day and risk factors. When an association was found, we examined whether the relationship between risk factors and cardiovascular mortality was affected by the adjustment for time of day using survival analyses.

Results: N-terminal pro-brain natriuretic peptide (NT-ProBNP) levels increased by 3.3% per hour [95% Confidence interval (CI) 1.9; 4.8], Interleukin-6 (IL-6) increased by 2.6% per hour (95% CI 1.8; 3.4), while Tissue plasminogen activator (t-PA) decreased by 3.3% per hour (95% CI 3.7; 2.9); these associations were unaffected by adjustment for possible confounding factors. The percentages of variation in these risk factors attributable to time of day were less than 2%. In survival analyses, the association of IL-6, NT-ProBNP, and t-PA with cardiovascular mortality was not affected by the adjustment for time of day. C-Reactive Protein, Fibrinogen, D-Dimer, von Willebrand Factor, and cardiac Troponin T showed no associations with time of day.

Conclusions: In older men, markers of inflammation (IL-6), haemostasis (t-PA), and a cardiac marker (NT-ProBNP) varied by time of day. The contribution of time of day to variations in these markers was small, and did not appear to be relevant for the CVD risk prediction.

For peer review only

Strengths and limitations of this study (Max 5 points)

1) In older adults diurnal variations in novel CVD risk factors have not been yet estimated; to our knowledge the findings from this study are novel

2) Previous studies suggested that diurnal variations in CVD risk factors could be relevant for cardiovascular risk prediction, but without testing the validity of this statement using statistical analysis. Our analysis answered this question and went beyond simple descriptive diurnal patterns of CVD risk factors

3) The BRHS cohort benefits from using a large scale population-based sample of free-living older men and this increases statistical power and precision of estimates.

4) However, the BRHS comprises male participants only, predominantly of white European ethnic origin, so findings may not be generalisable to women and non-white ethnic groups.

For peer review only

Background

Previous studies have reported time of day variation in both established and novel cardiovascular disease (CVD) risk factors in middle aged adults, such as blood pressure, lipids and some well-established inflammatory and haemostatic factors (e.g. white blood cell, red blood cell, and platelets counts) [1-3]. However, the extent to which novel CVD risk factors such as Interleukin-6, a marker of inflammation causally associated with CHD in a recent study [4], and N-terminal pro-brain natriuretic peptide, a promising marker of heart failure [5] vary by time of day have been less studied. Moreover, very little is known on time of day variations in other emerging risk factors prospectively associated with CVD (e.g. t-PA, D-Dimer, von Willebrand factor, and Cardiac Troponin T), although their causal association with CVD remain debated or not yet tested.

We would expect that time of day variations in some novel CVD risk factors measured in older adults may occur, consistent with findings in younger populations [6]. However, in older adults the degree of difference attributable to time of day has not been yet estimated; establishing its importance and its effects on prediction of CVD risk is important given the potentially wider use of N-terminal pro-brain natriuretic peptide in risk stratification (as shown in a recent major meta-analysis in the general population [7]), and potential causal link between IL-6 and cardiovascular disease [4]. Therefore, the aim of this study was to investigate how novel CVD risk factors, including markers of inflammation, haemostasis and myocardial function, varied by time of day in older British men.

Methods

Participants

The British Regional Heart Study (BRHS) is a prospective cohort study of cardiovascular disease involving 7735 middle aged men (40-59 years) selected in 1978-80 from the age-sex registers of one local primary care centre in 24 British towns [8]. The 24 towns were selected to represent the variation in cardiovascular disease across the UK [9]. The National Research Ethics Service (NRES) Committee for London provided ethical approval. Participants provided informed written consent to the investigation, which was performed in accordance with the Declaration of Helsinki [10].

For peer review only

Follow-up examination

In 1998-2000, an average of 20 years after the initial recruitment, 4252 surviving participants (77% response rate) aged 60-79 years who were resident in the UK attended a physical examination during which nurses took a fasting blood sample on one occasion for each participant.. The men were asked to fast for a minimum of 6 hours, during which they were instructed to drink only water, as previously reported [2]. The blood samples were collected between 08:00 h and 19:00 h and then assayed for a range of biochemical and haematological markers. The participants were also asked to complete a questionnaire which included questions on other established CVD risk factors, such as age, social class, smoking habits, alcohol consumption, and physical activity. Specifically, physical activity levels were self-reported [11] and recently validated using accelerometers [12]. Incident CVD, including non-fatal stroke and non-fatal MI were recorded: their definitions have been reported elsewhere [13]. Men were also asked whether a doctor had ever told them that they had heart failure [5]. The number of blood samples collected and included in the analyses differ according to the risk factor measurements (the number of observations varied from 3580 for N-terminal Pro-Brain Natriuretic Peptide to 3863 for von Willebrand Factor in complete case analyses including all covariates of interest).

Novel risk factors

Circulating levels of markers of inflammation (C-reactive protein [CRP], Interleukin 6 [IL-6], Fibrinogen), cardiac markers (N-terminal pro-brain natriuretic peptide [NT-ProBNP], cardiac Troponin T [cTnT], and markers of haemostasis (tissue plasminogen activator [t-PA] antigen, fibrin D-dimer, von Willebrand factor [vWF]) were measured.

Plasma D-dimer and t-PA levels were measured using an enzyme-linked immunosorbent assay (ELISA; Biopool AB, Umeå, Sweden), as was VWF antigen (Dako, High Wycombe, UK). C-reactive protein was assayed using ultrasensitive nephelometry (Dade Behring, Milton Keynes, UK). IL-6 was assayed using a high-sensitivity ELISA (R & D Sys-tems, Oxford, UK). Fibrinogen was assayed using an auto-mated Clauss assay in a coagulometer (MDA-180, Organon Teknika, Cambridge, UK). NT-proBNP and hsTnT were measured in plasma samples on an automated clinically validated immunoassay analyzer (e411, Roche Diagnostics,

For peer review only

Burgess Hill, United Kingdom) using the manufacturers' calibrators and quality control reagents. Intra- and inter-assay Coefficient of Variations (CVs) were, respectively: 4.1% and 6.6% for t-PA; 3.2% and 4.2% for vWF; 4.7% and 5.2% for D-dimer; 4.7% and 8.3% for CRP; 7.5% and 8.9% for IL-6; 2.6% and 3.7% for Fibrinogen, and 4.4% and 7.7% for NT-ProBNP and cTnT.

Statistical methods

Firstly, the distributions of the outcomes were examined; the outcomes were log-transformed as the distributions were positively skewed. Therefore, analysis was carried out on their log-transformed values throughout. Unadjusted geometric means and 95% Confidence Intervals [CI] of the outcomes were plotted against hour of the day.

Adjusted associations between time of day and the outcomes

Associations between time of day (fitted as a continuous variable, range 8-18) and the outcomes were examined using linear multilevel random intercept models (level 1 = individual, level 2 = town of residence). The results can be interpreted as between-person variations over the course of the examination day; the estimates from the linear model were reported as the difference in the outcome levels per hour of sampling over the examination day. As the outcomes were log-transformed, the results were reported as percent difference in the outcome geometric mean per hour of sampling. All models were initially adjusted for age only. Next, the models were adjusted for age and other possible confounding factors: social class, BMI, previous stroke or myocardial infarction (MI), physical activity, smoking status, alcohol consumption, use of statin, and a seasonal term (fitted using a cosinor function, as in previous studies) [14]. As NT-ProBNP and cTnT are principally markers of heart failure, the association with time of day was adjusted for previous heart failure.

When the association of time of the day with the outcomes was found to be statistically significant, the proportion of variance associated with time of the day was estimated using partial R-squared.

For peer review only

Sensitivity analyses

Five sensitivity analyses were performed: (i) all models were additionally adjusted for fasting time; (ii) interactions were fitted to test whether the time of day associations were modified by age (fitted as continuous variable); (iii) as NT-ProBNP and cTnT were acknowledged as specific cardiac markers [5], interactions were fitted to test whether the time of day associations were modified by previous heart failure (yes/no); (iv) to explore the potential of undiagnosed heart failure or cardiac damage influencing findings for NT-ProBNP and cTnT, we repeated regression models after excluding men with NTproBNP > 400 pg/ml; (v) a quadratic term for time of day was added to the models in order to check for non-linearity

As IL-6 has been causally associated with cardiovascular risk [4], and prospectively associated with CVD mortality in the BRHS sample used here [15], we investigated the relevance of time of day to the cardiovascular risk prediction by performing two survival analyses: in the first analysis we used Cox models where unadjusted log IL-6 was used as the predictor and CVD mortality as the clinical outcome; then, we repeated the same analysis using log IL-6 standardised by the time of day rather than unadjusted log IL-6. For completeness of information, we repeated this sensitivity analysis for NT-ProBNP and t-PA.

Results

The characteristics of the study participants (mean age 68.7 years, standard deviation (SD) = 5.5) are reported in Table 1. The associations between time of day (by hour) and risk factors are shown in Figure 1. Evidence of an increase over the course of the day was particularly noticeable for IL-6, and for NT-ProBNP (Figure 1). Also, levels of t-PA were lower in the afternoon in comparison with morning, while variations by time of day for other risk factors were not clearly observable from the plots (Figure 1). The results of corresponding linear regression analyses are shown in Table 2: statistically significant associations between time of the day and some outcomes were found (Table 2): over the course of the examination day NT-ProBNP levels increased by 3.3% per hour (95% CI 1.9; 4.8%), IL-6 increased by 2.6% per hour (95% CI 1.8; 3.4%). Conversely, t-PA decreased by 3.3% per hour (95% CI 3.7; 2.9%). The proportion of variance associated with time of the day from the fully adjusted models was 0.5%, 1%, and 2% for NT-ProBNP, IL-6, and t-PA respectively.

For peer review only

C-Reactive Protein, Fibrinogen, D-Dimer, von Willebrand Factor, and cardiac Troponin T showed no consistent associations with time of day (Table 2).

Sensitivity analyses

Overall, we found that fasting time did not alter the magnitude of associations between time of the day and the outcomes reported in Table 2. Only the association between time of the day and t-PA was strongly attenuated after accounting for fasting time (fitted as continuous variable): the decrease in t-PA levels was -3.3% (95%CI -3.7; -2.9) per hour before the adjustment (Table 2) and -1.4% (95%CI -2.2; -0.1) after the adjustment for fasting.

For all outcomes, we also did not find evidence for an interaction between of time of day with age (results not shown).

In stratified analysis, NT-ProBNP levels increased by 3.4% (95% CI 1.9; 4.8%, p<0.001) per hour in older men without heart failure. Although men who previously had heart failure had increased NT-ProBNP levels, there was no evidence for an interaction between previous heart failure with time of the day (p=0.954). After excluding 466 men with NTproBNP levels >400pg/ml (12% of the sample), associations between time of the day measures and NTproBNP remained statistically significant and slightly increased in magnitude (3.9% [95% CI 2.7; 5.1%], p<0.001). As reported in the main analysis, no significant associations were found between time of the day and cTnT in stratified analysis.

When adding a quadratic term to the model, we found a significant improvement in model fit for IL-6 only (p=0.030 for the time of day squared term). The association of time of day with IL-6 appeared to be slightly J-shaped, with a linear increase starting from 11:00 until 18:00 hours.

We examined whether adjustment for hour of day affected the associations between risk factors and CVD mortality. In survival analysis, higher levels of log IL-6 were associated with increased CVD mortality (HR=1.70, 95%CI 1.54; 1.87). Standardising IL-6 by time of the day did not change the relationship (HR=1.71, 95%CI 1.55; 1.88). Also, standardising NT-ProBNP

For peer review only - http://bmjopen.bmj.com/site/about/guidelines.xhtml

levels by time of the day did not alter the magnitude of the effect on CVD mortality (HR=1.92, 95%CI 1.81; 2.04). Finally, associations of t-PA levels with increased CVD mortality did not change substantially before (HR=1.74, 95%CI 1.45; 2.09) and after standardising (HR=1.77, 95%CI 1.47; 2.14) by time of the day.

Discussion

To our knowledge, this is the largest investigation of relationships between time of day and novel CVD risk factors in older men. After adjusting our analysis for potential confounding factors we demonstrated that some, but not all, novel CVD risk factors levels varied by time of day. In particular, NT-ProBNP and IL-6 increased linearly over the course of the day. Conversely, a decrease in t-PA was also observed; however, after accounting for fasting time the relationship with time of the day was strongly attenuated (therefore fasting time could partially explain the drop in t-PA levels observed in the afternoon vs morning). Also, we observed a weak contribution of time of the day to the overall variation of these markers. In sensitivity analyses, we observed that time of day did not have a sufficiently strong effect to be taken into account when assessing the impact of IL-6, NT-ProBNP, and t-PA on CVD mortality. Lastly, an association of time of day with other novel risk factors was not observed.

Literature on time of day variation in novel CVD markers of inflammation and haemostasis in older adults is limited; to our knowledge this is the first time these findings have been reported in older adults. Findings from earlier studies of younger adults were fairly consistent with ours. For example a recent meta-analysis of several small studies which analysed IL-6 proposed a diurnal pattern, with overall IL-6 levels increased between 08:00 and 18:00 hours as in our study [16]. However, in two previous very small studies of twelve [17] and five [18] participants, IL-6 peaked in the night-time. It is possible that peaks in IL-6 levels may be associated with cognitive symptoms of depression [19] and daily activities, although in the BRHS population this has not yet been investigated. One previous study found that BRHS men were more active in the morning and in early afternoon [20] when the main activities were usually gardening, house works, shopping, or leisure walking. Whether IL-6 was

For peer review only

implicated in this daily pattern remains uncertain and can potentially be explored in future studies.

Moreover, one previous study reported increased levels of NT-ProBNP over the course of day [21] as we observed in our study. A decrease in t-PA over the examination day was also reported in younger subjects (a 45-year-old UK population of 9377 men and women) [6]; however, t-PA did not vary by time of the day in a previous study large study of 1288 healthy 25 to 64-year-old men and women [22].

In comparison to our study, findings regarding CRP, Fibrinogen, D-dimer, vWF and cTnT reported in earlier studies of younger adults were similar: a few previous studies reported that they did not find an association of time of day with CRP [23], D-dimer [24], and vWF [25]. In one study, the variation in CRP, Fibrinogen, D-dimer, and vWF attributable to time of day was minimal [6]. Literature on cTnT is scarce: one small previous study of repeated measures in 7 participants with type 2 diabetes reported a decrease in cTnT between 8am and 8pm [26].

Although one previous study suggested that diurnal variations in CVD risk factors could be relevant for cardiovascular risk prediction [6], a prediction model like the one described in our survival analysis was not performed. Our findings suggested the effect of time of the day (from 08:00 h to 19:00 h) is not relevant for the CVD risk assessment. With this sensitivity analysis we wanted to investigate time of day variations beyond simple descriptive diurnal patterns; to our knowledge this is the first time this finding has been reported.

Strengths and limitations

The BRHS cohort benefits from using a large scale population-based sample of free-living older men and this increases statistical power and precision of estimates. However, the BRHS comprises male participants, predominantly of white European ethnic origin, so findings may not be generalisable to women and non-white ethnic groups. The CVD risk factors measurements were carried out on one occasion over an extended period of the day (between 08:00 and 18:00 hours), offering only a partial understanding of the variations over the 24 hours [27 28]. Therefore, in this study the relationship of the CVD risk factors to time of day was explored using between-participant variation only. In future studies it may

For peer review only

be advantageous to carry out repeated measurement of the risk factors over the 24 hours in order to investigate within-person circadian variations. However, with repeated measurements a possible and genuine diurnal variation may be disrupted and natural sleeping patterns altered (repeated measures are usually taken every 1-2 hours during the night) [29].

Implications

Variations of some CVD factors (in particular IL-6 and NT-ProBNP) over the course of the day were observed, suggesting the role of time of the day as potential confounder during the measurements. However, standardising these biological markers by time of day was not particularly relevant for the cardiovascular risk prediction. Also, other sensitivity analyses (stratified analysis and interaction tests) did not add relevant insights suggesting that time of day variations may be not important for clinical risk stratification in general. Further studies assessing both CVD risk factors levels and clinical outcomes (e.g. fatal or non-fatal CVD events) during 24h are required to demonstrate whether a rapid increase of IL-6 over the day may be relevant to the increased number of CVD events observed in early and late morning [30], and whether the increased levels of NT-ProBNP over the day are related to the afternoon peak in sudden death following heart failure [31].

Conclusions

Variations in time of day were associated with variations of some, but not all, novel CVD risk factors measured in older adults. The contribution of time of the day to the markers' overall variation was small and unlikely to affect the CVD risk prediction or clinical risk stratification.

AUTHORS CONTRIBUTIONS

CS processed the data, performed statistical analyses, drafted and revised the manuscript, and incorporated revisions of co-authors. PHW, SGW, BJ, and RM contributed to the design of the study and revised the manuscript. LL enrolled participants, and collected data. PHW, RM and SGW raised grant funding. All authors gave an intellectual contribution to the manuscript and approved the final version.

Acknowledgements

For peer review only

The BRHS is supported by a British Heart Foundation (BHF) programme grant (RG/13/16/30528). This research was supported by a BHF project grant (PG/13/41/30304) which supported CS. The funders had no role in the design and conduct of the study; collection, management, analysis, and interpretation of the data; preparation, review, or approval of the manuscript; and the decision to submit the manuscript for publication. The views expressed in this publication are those of the author(s) and not necessarily those of the BHF.

Conflict of interest statement

The authors report no relationships that could be construed as a conflict of interest

Data sharing statement

The collection and management of data over the last 39 years of the BRHS has been made possible through grant funding from UK government agencies and charities. We welcome proposals for collaborative projects and data sharing. For general data sharing enquiries, please contact Lucy Lennon l.lennon@ucl.ac.uk

For peer review only

323 **Table 1** –Individual characteristics and risk factors levels in BRHS men who attended the
324 examinations in 1998-2000

Demographic and background characteristics	
Age (years), mean (SD)	68.7 (5.5)
Social class (manual)	
Manual, n (%)	2166 (51.1)
Non-Manual, n (%)	1966 (46.3)
HMF, n (%)	112 (2.6)
Physical health	
BMI, mean (SD)	26.9 (3.7)
Prevalence of stroke/myocardial infarction, n (%)	153 (3.6)
Prevalence of heart failure, n (%)	390 (9.2)
Behavioural factors	
Smoking	
Never, n (%)	1233 (29.1)
Ex-smokers, n (%)	2464 (58.0)
Smokers, n (%)	548 (12.9)
Alcohol consumption	
None, n (%)	431 (10.3)
Occasional/light, n (%) [1]	2949 (70.5)
Moderate/Heavy, n (%) [2]	779 (18.6)
Physical activity level	
Inactive, n (%)	471 (11.5)
Occasionally, n (%)	957 (23.4)
Light, n (%)	767 (18.7)
Moderate, n (%)	591 (14.4)
Moderate vigorous, n (%)	690 (16.8)
Vigorous, n (%)	621 (15.1)
CVD risk factor, geometric mean (SD)	
CRP, mg/L	1.74 (3.03)
IL-6, pg/mL	2.46 (1.94)
Fibrinogen, g/L	3.19 (1.25)
t-PA, ng/mL	10.23 (1.50)
vWF, IU/dL	132.41 (1.40)
D-dimer, ng/mL	84.32 (2.32)
NT-ProBNP, pg/mL	101.50 (3.32)
cTnT, pg/mL	12.07 (1.64)

325

326 [1] >=1 and <=15 units per week (1 unit is approximately 1 drink, such as one glass of wine)
327 [2] >=16 units per week (1 unit is approximately 1 drink, such as one glass of wine)

328

For peer review only

Table 2 – Cross-sectional adjusted associations between time of day (fitted as continuous variable) and CVD risk factors measured in BRHS men (aged 60-79) attending the follow-up year 20 examination in 1998-2000. Associations are reported as percent difference in CVD risk factors levels per one hour of sampling over the examination day (08:00-19:00h). The statistically significant associations are marked in bold.

	Model 1: Age adjusted [1]		Model 2: Fully adjusted [2]	
CVD risk factor	Percent difference (95%CI) in the CVD risk factor levels per hour of sampling [3]	p-value	Percent difference (95%CI) in the CVD risk factor levels per hour of sampling [3]	p-value
NT-ProBNP	3.5 (2.0;5.0)	<0.001	3.3 (1.9; 4.8)	<0.001
IL-6	2.6 (1.7;3.4)	<0.001	2.6 (1.8; 3.4)	<0.001
t-PA	-3.3 (-3.8;-2.9)	<0.001	-3.3 (-3.7; -2.9)	<0.001
Fibrinogen	-0.2 (-0.5;0.0)	0.088	-0.2 (-0.5; 0.1)	0.104
cTnT	-0.4 (-0.9;0.2)	0.194	-0.4 (-1.0; 0.2)	0.174
CRP	-1.0 (-2.3;0.4)	0.151	-0.9 (-2.2; 0.4)	0.175
vWF	-0.2 (-0.6;0.2)	0.374	-0.2 (-0.6; 0.2)	0.380
D-Dimer	-0.0 (-1.0;1.0)	0.929	-0.1 (-1.0; 0.9)	0.890

[1] Model 1: Two level linear models (level 1 = person, level 2 = town of residence during the BRHS recruitment) adjusted for age. Model 1 used the same number of observations of Model 2 (complete case analysis).

[2] Model 2 = Model 1 additionally adjusted for social class, BMI, smoking status, alcohol consumption, physical activity, use of statin, and season. Associations with IL-6, t-PA, Fibrinogen, CRP, vWF, and D-Dimer were additionally adjusted for prevalence of stroke/MI, while association of time of the day with NT-ProBNP and cTnT models were additionally adjusted for prevalence of heart failure.

[3] Model 1 and Model 2 used the number of observations: 3580 for NT-ProBNP, 3832 for IL-6, 3863 for t-PA, 3861 for Fibrinogen, 3827 for cTnT, 3838 for CRP, 3863 for vWF, 3859 for D-Dimer

For peer review only

Figure 1 – Unadjsted geometric means (95% CI) by time of the day [1] for CRP, IL-6, Fibrinogen t-PA (top), vWF, D-Dimer, NT-ProBNP and cTnT (bottom) measured on one occasion in BRHS men aged 60-79 during the years 1998-2000.

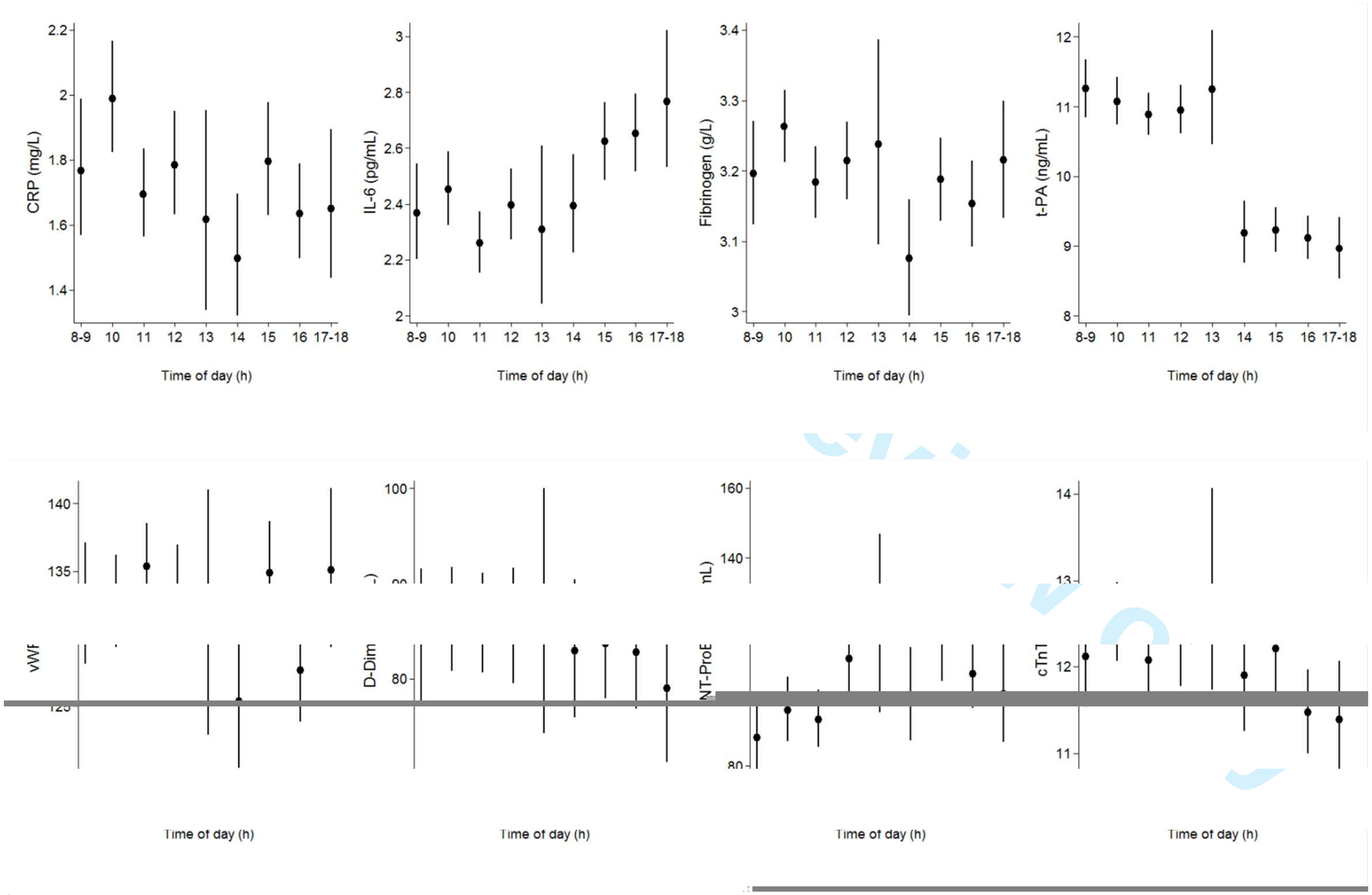

[1] Total number of men examined per hour was 33(0.7%) at 08:00-08:59, 363(8.5%) at 8:00-9:59h, 699(16%) at 10:00-10:59h, 771(18%) at 11:00-11:59h, 591(14%) at 12:00-12:59h, 99(2%) at 13:00-13:59h, 306(7%) at 14:00-14:59), 560(13%) at 15:00-15:59h, 566(13%) at 16:00-16:59, 260(6%) at 17:00-17:59, and 3(<0.1%) at 18:00-18:59

References

1. Pocock SJ, Ashby D, Shaper AG*, et al.* Diurnal variations in serum biochemical and haematological measurements. *Journal of clinical pathology*. 1989; 42: 172-9.
2. Emberson JR, Whincup PH, Walker M*, et al.* Biochemical measures in a population-based study: effect of fasting duration and time of day. *Annals of clinical biochemistry*. 2002; 39: 493-501.
3. Elliott WJ. Circadian variation in blood pressure: Implications for the elderly patient. *American Journal of Hypertension*. 1999; 12: 43S-9S.
4. IL6R Genetics Consortium and Emerging Risk Factors Collaboration. Interleukin-6 receptor pathways in coronary heart disease: a collaborative meta-analysis of 82 studies. *The Lancet*. 2012; 379: 1205-13.
5. Wannamethee SG, Shaper AG, Papacosta O*, et al.* Lung function and airway obstruction: associations with circulating markers of cardiac function and incident heart failure in older men-the British Regional Heart Study. *Thorax*. 2016; 71: 526-34.
6. Rudnicka AR, Rumley A, Lowe GD*, et al.* Diurnal, seasonal, and blood-processing patterns in levels of circulating fibrinogen, fibrin D-dimer, C-reactive protein, tissue plasminogen activator, and von Willebrand factor in a 45-year-old population. *Circulation*. 2007; 115: 996-1003.
7. Natriuretic Peptides Studies Collaboration. Natriuretic peptides and integrated risk assessment for cardiovascular disease: an individual-participant-data meta-analysis. *The lancet Diabetes & endocrinology*. 2016; 4: 840-9.
8. Shaper AG, Pocock SJ, Walker M*, et al.* British Regional Heart Study: cardiovascular risk factors in middle-aged men in 24 towns. *BMJ*. 1981; 283: 179-86.
9. Pocock SJ, Shaper AG, Cook DG*, et al.* British Regional Heart Study: geographic variations in cardiovascular mortality, and the role of water quality. *British Medical Journal*. 1980; 280: 1243-9.
10. Declaration of Helsinki - Ethical Principles for Medical Research Involving Human Subjects. *WMA Declaration of Helsinki*. WMA Declaration of HelsinkiOctober 2013.
11. Wannamethee G and Shaper AG. Physical activity and stroke in British middle aged men. *BMJ : British Medical Journal*. 1992; 304: 597-601.
12. Jefferis BJ, Sartini C, Ash S*, et al.* Validity of questionnaire-based assessment of sedentary behaviour and physical activity in a population-based cohort of older men; comparisons with objectively measured physical activity data. *Int J Behav Nutr Phys Act*. 2016; 13: 14.
13. Sartini C, Barry SJ, Wannamethee SG*, et al.* Effect of cold spells and their modifiers on cardiovascular disease events: Evidence from two prospective studies. *Int J Cardiol*. 2016; 218: 275-83.
14. Stolwijk AM, Straatman H and Zielhuis GA. Studying seasonality by using sine and cosine functions in regression analysis. *Journal of Epidemiology and Community Health*. 1999; 53: 235-8.
15. Wannamethee SG, Whincup PH, Lennon L*, et al.* Associations Between Fibrin D-Dimer, Markers of Inflammation, Incident Self-Reported Mobility Limitation, and All-Cause Mortality in Older Men. *Journal of the American Geriatrics Society*. 2014; 62: 2357-62.
16. Nilsonne G, Lekander M, Åkerstedt T*, et al.* Diurnal variation of circulating interleukin-6 in humans: a meta-analysis. *bioRxiv*. 2016.

For peer review only

17. Gudewill S, Pollmacher T, Vedder H*, et al.* Nocturnal plasma levels of cytokines in healthy men. *European archives of psychiatry and clinical neuroscience*. 1992; 242: 53-6.
18. Bauer J, Hohagen F, Ebert T*, et al.* Interleukin-6 serum levels in healthy persons correspond to the sleep-wake cycle. *The Clinical investigator*. 1994; 72: 315-.
19. Gimeno D, Kivimaki M, Brunner EJ*, et al.* Associations of C-reactive protein and interleukin-6 with cognitive symptoms of depression: 12-year follow-up of the Whitehall II study. *Psychological medicine*. 2009; 39: 413-23.
20. Sartini C, Wannamethee SG, Iliffe S*, et al.* Diurnal patterns of objectively measured physical activity and sedentary behaviour in older men. *BMC Public Health*. 2015; 15: 609.
21. Goetze JP, Jorgensen HL, Sennels HP*, et al.* Diurnal plasma concentrations of natriuretic propeptides in healthy young males. *Clinical chemistry*. 2012; 58: 789-92.
22. Eliasson M, Evrin PE, Lundblad D*, et al.* Influence of gender, age and sampling time on plasma fibrinolytic variables and fibrinogen. *Fibrinolysis*. 1993; 7: 316-23.
23. Meier-Ewert HK, Ridker PM, Rifai N*, et al.* Absence of diurnal variation of C-reactive protein concentrations in healthy human subjects. *Clinical chemistry*. 2001; 47: 426-30.
24. Jafri SM, VanRollins M, Ozawa T*, et al.* Circadian variation in platelet function in healthy volunteers. *Am J Cardiol*. 1992; 69: 951-4.
25. Bridges AB, McLaren M, Scott NA*, et al.* Circadian variation of tissue plasminogen activator and its inhibitor, von Willebrand factor antigen, and prostacyclin stimulating factor in men with ischaemic heart disease. *British Heart Journal*. 1993; 69: 121-4.
26. Klinkenberg LJ, van Dijk JW, Tan FE*, et al.* Circulating cardiac troponin T exhibits a diurnal rhythm. *Journal of the American College of Cardiology*. 2014; 63: 1788-95.
27. Bremner WF, Sothern RB, Kanabrocki EL*, et al.* Relation between circadian patterns in levels of circulating lipoprotein(a), fibrinogen, platelets, and related lipid variables in men. *American heart journal*. 2000; 139: 164-73.
28. Kristal-Boneh E, Harari G and Green MS. Seasonal Change in 24-Hour Blood Pressure and Heart Rate Is Greater Among Smokers Than Nonsmokers. *Hypertension*. 1997; 30: 436-41.
29. Vgontzas AN, Zoumakis M, Bixler EO*, et al.* Impaired nighttime sleep in healthy old versus young adults is associated with elevated plasma interleukin-6 and cortisol levels: physiologic and therapeutic implications. *J Clin Endocrinol Metab*. 2003; 88: 2087-95.
30. Elliott WJ. Circadian variation in the timing of stroke onset: a meta-analysis. *Stroke; a journal of cerebral circulation*. 1998; 29: 992-6.
31. Carson PA, O'Connor CM, Miller AB*, et al.* Circadian rhythm and sudden death in heart failureResults from prospective randomized amlodipine survival trial. *Journal of the American College of Cardiology*. 2000; 36: 541-6.

For peer review only

STROBE Statement—Checklist of items that should be included in reports of ***cross-sectional studies***

	Item No	Recommendation	
**Title and abstract**	1	(*a*) Indicate the study's design with a commonly used term in the title or the abstract	Abstract
		(*b*) Provide in the abstract an informative and balanced summary of what was done and what was found	Abstract
**Introduction**			
Background/rationale	2	Explain the scientific background and rationale for the investigation being reported	Introduction, lines 66-68
Objectives	3	State specific objectives, including any prespecified hypotheses	Introduction, lines 66-67 72-74
**Methods**			
Study design	4	Present key elements of study design early in the paper	Methods, line 78-81
Setting	5	Describe the setting, locations, and relevant dates, including periods of recruitment, exposure, follow-up, and data collection	Methods, lines 86-93
Participants	6	(*a*) Give the eligibility criteria, and the sources and methods of selection of participants	Methods, lines 78-81 and 86-90
Variables	7	Clearly define all outcomes, exposures, predictors, potential confounders, and effect modifiers. Give diagnostic criteria, if applicable	Methods , lines 104-121
Data sources/ measurement	8*	For each variable of interest, give sources of data and details of methods of assessment (measurement). Describe comparability of assessment methods if there is more than one group	Methods , lines 91-121
Bias	9	Describe any efforts to address potential sources of bias	
Study size	10	Explain how the study size was arrived at 4252	Results, lines 86-90
Quantitative variables	11	Explain how quantitative variables were handled in the analyses. If applicable, describe which groupings were chosen and why	Methods , lines 104-121
Statistical methods	12	(*a*) Describe all statistical methods, including those used to control for confounding	Methods , lines 123-166
		(*b*) Describe any methods used to examine subgroups and interactions	Methods , lines 150-166
		(*c*) Explain how missing data were addressed	
		(*d*) If applicable, describe analytical methods taking account of sampling strategy	NA
		(*e*) Describe any sensitivity analyses	Methods , lines 150-166
**Results**			
Participants	13*	(a) Report numbers of individuals at each stage of study—eg numbers potentially eligible, examined for eligibility, confirmed eligible, included in the study, completing follow-up, and analysed	Methods, lines 78-81 and 86-90
		(b) Give reasons for non-participation at each stage	Methods, lines 78-81 and 86-90
		(c) Consider use of a flow diagram	Not necessary for this study, see Methods, lines 78-81 and 86-90

For peer review only

Descriptive data	14*	(a) Give characteristics of study participants (eg demographic, clinical, social) and information on exposures and potential confounders	Table 1
		(b) Indicate number of participants with missing data for each variable of interest	Table 1
Outcome data	15*	Report numbers of outcome events or summary measures	Table 1, Figure 1
Main results	16	(*a*) Give unadjusted estimates and, if applicable, confounder-adjusted estimates and their precision (eg, 95% confidence interval). Make clear which confounders were adjusted for and why they were included	Table 2
		(*b*) Report category boundaries when continuous variables were categorized	NA
		(*c*) If relevant, consider translating estimates of relative risk into absolute risk for a meaningful time period	NA
Other analyses	17	Report other analyses done—eg analyses of subgroups and interactions, and sensitivity analyses	Results, lines 195-216
**Discussion**			
Key results	18	Summarise key results with reference to study objectives	Results, lines 169-180 Discussion, lines 219-230
Limitations	19	Discuss limitations of the study, taking into account sources of potential bias or imprecision. Discuss both direction and magnitude of any potential bias	Discussion, lines lines 267-279
Interpretation	20	Give a cautious overall interpretation of results considering objectives, limitations, multiplicity of analyses, results from similar studies, and other relevant evidence	Discussion, lines 232-244 and 282 - 292
Generalisability	21	Discuss the generalisability (external validity) of the study results	Discussion, lines 295-297
**Other information**			
Funding	22	Give the source of funding and the role of the funders for the present study and, if applicable, for the original study on which the present article is based	Acknowledgements, lines 306-314

*Give information separately for exposed and unexposed groups.

Note: An Explanation and Elaboration article discusses each checklist item and gives methodological background and published examples of transparent reporting. The STROBE checklist is best used in conjunction with this article (freely available on the Web sites of PLoS Medicine at http://www.plosmedicine.org/, Annals of Internal Medicine at http://www.annals.org/, and Epidemiology at http://www.epidem.com/). Information on the STROBE Initiative is available at www.strobe-statement.org.

For peer review only

BMJ Open

Time of day variations in cardiovascular disease risk factors measured in older men

Journal:	*BMJ Open*
Manuscript ID	bmjopen-2017-018264.R1
Article Type:	Research
Date Submitted by the Author:	18-Aug-2017
Complete List of Authors:	Sartini, Claudio; University College of London, Primary Care and Population Health<br>Whincup, Peter; St Georges, University of London, Population Health Research Institute<br>Wannamethee, Goya; University College London<br>Jefferis, Barbara ; University College London<br>Lennon, Lucy; UCL, Department of Primary Care and Population Health<br>Lowe, Gordon; University of Glasgow, Institute of Cardiovascular and Medical Sciences<br>Welsh, Paul; University of Glasgow, Institute of Cardiovascular and Medical Sciences<br>Sattar, Naveed; University of Glasgow, BHF centre<br>Morris, Richard; University of Bristol, School of Social & Community Medicine
**Primary Subject Heading**:	Epidemiology
Secondary Subject Heading:	Public health
Keywords:	biological markers, older adults, cardiovascular disease, diurnal variations

SCHOLARONE™
Manuscripts

Title: Time of day variations in cardiovascular disease risk factors measured in older men

Claudio Sartini, MSc [1 *], Peter H Whincup, PhD [2], S Goya Wannamethee, PhD [1], Barbara J Jefferis, PhD [1], Lucy Lennon, MSc [1], Gordon DO Lowe, PhD [3], Paul Welsh, PhD [3], Naveed Sattar, PhD [3], and Richard W Morris, PhD [4]

[1] Department of Primary Care & Population Health, University College London, Rowland Hill Street, NW3 2PF, London. UK

[2] Population Health Research Institute, St George's University of London, Cranmer Terrace London SW17 0RE, UK.

[3] Institute of Cardiovascular and Medical Sciences, University of Glasgow, Glasgow G12 8TA UK

[4] School of Social & Community Medicine, University of Bristol, Canynge Hall, 39 Whatley Rd, Bristol BS8 2PS, UK.

[*] Corresponding Author

E-mail: c.sartini@ucl.ac.uk;

Manuscript number of words: 4847 words (all included: title page, abstract, manuscript, all tables and references).

For peer review only

Abstract (250 words, now 248)

Objective: We estimated associations of time of day with cardiovascular disease (CVD) risk factors measured in older men.

Methods: CVD risk factors (markers of inflammation and haemostasis, and cardiac markers) were measured on one occasion between 08:00-19:00 hours in 4252 men aged 60-79 years from the British Regional Heart Study. Linear models were used to estimate associations between time of day and risk factors. When an association was found, we examined whether the relationship between risk factors and cardiovascular mortality was affected by the adjustment for time of day using survival analyses.

Results: N-terminal pro-brain natriuretic peptide (NT-ProBNP) levels increased by 3.3% per hour [95% Confidence interval (CI) 1.9; 4.8], Interleukin-6 (IL-6) increased by 2.6% per hour (95% CI 1.8; 3.4), while Tissue plasminogen activator (t-PA) decreased by 3.3% per hour (95% CI 3.7; 2.9); these associations were unaffected by adjustment for possible confounding factors. The percentages of variation in these risk factors attributable to time of day were less than 2%. In survival analyses, the association of IL-6, NT-ProBNP, and t-PA with cardiovascular mortality was not affected by the adjustment for time of day. C-Reactive Protein, Fibrinogen, D-Dimer, von Willebrand Factor, and cardiac Troponin T showed no associations with time of day.

Conclusions: In older men, markers of inflammation (IL-6), haemostasis (t-PA), and a cardiac marker (NT-ProBNP) varied by time of day. The contribution of time of day to variations in these markers was small, and did not appear to be relevant for the CVD risk prediction.

For peer review only

Background

Previous studies have reported time of day variation in both established and emerging cardiovascular disease (CVD) risk factors in middle aged adults, such as blood pressure, lipids and some well-established inflammatory and haemostatic factors (e.g. white blood cell, red blood cell, and platelets counts) [1-3]. However, the extent to which emerging CVD risk factors such as Interleukin-6, a marker of inflammation causally associated with CHD in a recent study [4], and N-terminal pro-brain natriuretic peptide, a marker of heart failure [5] vary by time of day have been less studied. Moreover, very little is known on time of day variations in other emerging risk factors prospectively associated with CVD (e.g. t-PA, D-Dimer, von Willebrand factor, and Cardiac Troponin T), although their causal association with CVD remain debated or not yet tested.

We would expect that time of day variations in some emerging CVD risk factors measured in older adults may occur, consistent with findings in younger populations [6]. However, in older adults the degree of difference attributable to time of day has not been yet estimated; establishing its importance and its effects on prediction of CVD risk is important given the potentially wider use of N-terminal pro-brain natriuretic peptide in risk stratification (as shown in a recent major meta-analysis in the general population [7]), and potential causal link between IL-6 and cardiovascular disease [4]. Therefore, the aim of this study was to investigate how emerging CVD risk factors, including markers of inflammation, haemostasis and myocardial function, varied by time of day in older British men.

Methods

Participants

The British Regional Heart Study (BRHS) is a prospective cohort study of cardiovascular disease involving 7735 middle aged men (40-59 years) selected in 1978-80 from the age-sex registers of one local primary care centre in 24 British towns [8]. The 24 towns were selected to represent the variation in cardiovascular disease across the UK [9]. The National Research Ethics Service (NRES) Committee for London provided ethical approval. Participants provided informed written consent to the investigation, which was performed in accordance with the Declaration of Helsinki [10].

For peer review only

Follow-up examination

In 1998-2000, an average of 20 years after the initial recruitment, 4252 surviving participants (77% response rate) aged 60-79 years who were resident in the UK attended a physical examination during which nurses took a fasting blood sample on one occasion for each participant. The men were asked to fast for a minimum of 6 hours, during which they were instructed to drink only water, as previously reported [2]. The blood samples were collected between 08:00 h and 19:00 h and then assayed for a range of biochemical and haematological markers. Participants' appointment times were non-systematically allocated. They were offered the opportunity to contact the BRHS team and change the time of examination, if unable to attend; a small proportion of participants did so.

The participants were also asked to complete a questionnaire which included questions on other established CVD risk factors, such as age, social class, smoking habits, alcohol consumption, and physical activity. Specifically, physical activity levels were self-reported [11] and recently validated using accelerometers [12]. Incident CVD, including non-fatal stroke and non-fatal MI were recorded: their definitions have been reported elsewhere [13]. Men were also asked whether a doctor had ever told them that they had heart failure [5]. The number of blood samples collected and included in the analyses differ according to the risk factor measurements (the number of observations varied from 3580 for N-terminal Pro-Brain Natriuretic Peptide to 3863 for von Willebrand Factor in complete case analyses including all covariates of interest).

CVD Risk factors

Circulating levels of markers of inflammation (C-reactive protein [CRP], Interleukin 6 [IL-6], Fibrinogen), cardiac markers (N-terminal pro-brain natriuretic peptide [NT-ProBNP], cardiac Troponin T [cTnT], and markers of haemostasis (tissue plasminogen activator [t-PA] antigen, fibrin D-dimer, von Willebrand factor [vWF]) were measured.

D-dimer and t-PA levels were measured using an enzyme-linked immunosorbent assay (ELISA; Biopool AB, Umeå, Sweden), as was VWF antigen (Dako, High Wycombe, UK). C-reactive protein was assayed using ultrasensitive nephelometry (Dade Behring, Milton Keynes, UK). IL-6 was assayed using a high-sensitivity ELISA (R & D Sys-tems, Oxford, UK).

For peer review only

Fibrinogen was assayed using an auto-mated Clauss assay in a coagulometer (MDA-180, Organon Teknika, Cambridge, UK). NT-proBNP and hsTnT were measured in plasma samples on an automated clinically validated immunoassay analyzer (e411, Roche Diagnostics, Burgess Hill, United Kingdom) using the manufacturers' calibrators and quality control reagents. Intra- and inter-assay Coefficient of Variations (CVs) were, respectively: 4.1% and 6.6% for t-PA; 3.2% and 4.2% for vWF; 4.7% and 5.2% for D-dimer; 4.7% and 8.3% for CRP; 7.5% and 8.9% for IL-6; 2.6% and 3.7% for Fibrinogen, and 4.4% and 7.7% for NT-ProBNP and cTnT.

The samples were centrifuged and separated on the morning or afternoon of collection and stored on site at -20°C until they were transferred to a central freezer storage location at -70°C within 2 weeks of sample collection. Samples were then transferred on dry ice to a single central laboratory and were thawed immediately before analysis. Plasma samples were used for all the analyses reported here. The original sample collection took place between January 1998 and March 2000. Most of the analyses described here were carried out during 2000, after a maximum of 3 years storage; NT-ProBNP and cTNT were analysed in 2009.

Statistical methods

Firstly, the distributions of the outcomes were examined; the outcomes were log-transformed as the distributions were positively skewed. Therefore, analysis was carried out on their log-transformed values throughout. Unadjusted geometric means and 95% Confidence Intervals [CI] of the outcomes were plotted against hour of the day.

Adjusted associations between time of day and the outcomes

Associations between time of day (fitted as a continuous variable, range 8-18) and the outcomes were examined using linear multilevel random intercept models (level 1 = individual, level 2 = town of residence). The results can be interpreted as between-person variations over the course of the examination day; the estimates from the linear model were reported as the difference in the outcome levels per hour of sampling over the examination day. As the outcomes were log-transformed, the results were reported as percent difference in the outcome geometric mean per hour of sampling. All models were

For peer review only

initially adjusted for age only. Next, the models were adjusted for age and other possible confounding factors: social class, BMI, previous stroke or myocardial infarction (MI), physical activity, smoking status, alcohol consumption, use of statin, and a seasonal term (fitted using a cosinor function, as in previous studies) [14]. As NT-ProBNP and cTnT are principally markers of heart failure, the association with time of day was adjusted for previous heart failure.

When the association of time of the day with the outcomes was found to be statistically significant, the proportion of variance associated with time of the day was estimated using partial R-squared.

Sensitivity analyses

Six sensitivity analyses were performed: (i) all models were additionally adjusted for fasting time and diabetes; (ii) all models were carried out excluding men with diabetes, (iii) interactions were fitted to test whether the time of day associations were modified by age (fitted as continuous variable); (iv) as NT-ProBNP and cTnT were acknowledged as specific cardiac markers [5], interactions were fitted to test whether the time of day associations were modified by previous heart failure (yes/no); (v) to explore the potential of undiagnosed heart failure or cardiac damage influencing findings for NT-ProBNP and cTnT, we repeated regression models after excluding men with NTproBNP > 400 pg/ml; (vi) a quadratic term for time of day was added to the models in order to check for non-linearity

As IL-6 has been causally associated with cardiovascular risk [4], and prospectively associated with CVD mortality in the BRHS sample used here [15], we investigated the relevance of time of day to the cardiovascular risk prediction by performing two survival analyses: in the first analysis we used Cox models where unadjusted log IL-6 was used as the predictor and CVD mortality as the clinical outcome; then, we repeated the same analysis using log IL-6 standardised by the time of day rather than unadjusted log IL-6. For completeness of information, we repeated this sensitivity analysis for NT-ProBNP and t-PA.

Results

The characteristics of the study participants (mean age 68.7 years, standard deviation (SD) =

For peer review only

5.5) are reported in Table 1. The associations between time of day (by hour) and risk factors are shown in Figure 1. Evidence of an increase over the course of the day was particularly noticeable for IL-6, and for NT-ProBNP (Figure 1). Also, levels of t-PA were lower in the afternoon in comparison with morning, while variations by time of day for other risk factors were not clearly observable from the plots (Figure 1). The results of corresponding linear regression analyses are shown in Table 2: statistically significant associations between time of the day and some outcomes were found (Table 2): over the course of the examination day NT-ProBNP levels increased by 3.3% per hour (95% CI 1.9; 4.8%), IL-6 increased by 2.6% per hour (95% CI 1.8; 3.4%). Conversely, t-PA decreased by 3.3% per hour (95% CI 3.7; 2.9%). The proportion of variance associated with time of the day from the fully adjusted models was 0.5%, 1%, and 2% for NT-ProBNP, IL-6, and t-PA respectively.

C-Reactive Protein, Fibrinogen, D-Dimer, von Willebrand Factor, and cardiac Troponin T showed no consistent associations with time of day (Table 2).

Sensitivity analyses

Overall, we found that fasting time did not alter the magnitude of associations between time of the day and the outcomes reported in Table 2. Only the association between time of the day and t-PA was strongly attenuated after accounting for fasting time (fitted as continuous variable): the decrease in t-PA levels was -3.3% (95%CI -3.7; -2.9) per hour before the adjustment (Table 2) and -1.4% (95%CI -2.2; -0.1) after the adjustment for fasting. An additional adjustment for diabetes status did not alter the magnitude of the association between hour of the day and the outcomes. We also performed the analysis excluding men with diabetes completely (Table 2 – Model 3), but the association between time of day and the outcomes did not substantially change.

For all outcomes, we also did not find evidence for an interaction between of time of day with age (results not shown).

In stratified analysis, NT-ProBNP levels increased by 3.4% (95% CI 1.9; 4.8%, $p<0.001$) per hour in older men without heart failure. Although men who previously had heart failure had increased NT-ProBNP levels, there was no evidence for an interaction between previous

For peer review only

heart failure with time of the day (p=0.954). After excluding 466 men with NTproBNP levels >400pg/ml (12% of the sample), associations between time of the day measures and NTproBNP remained statistically significant and slightly increased in magnitude (3.9% [95% CI 2.7; 5.1%], p<0.001). As reported in the main analysis, no significant associations were found between time of the day and cTnT in stratified analysis.

When adding a quadratic term to the model, we found a significant improvement in model fit for IL-6 only (p=0.030 for the time of day squared term). The association of time of day with IL-6 appeared to be slightly J-shaped, with a linear increase starting from 11:00 until 19:00 hours.

We examined whether adjustment for hour of day affected the associations between risk factors and CVD mortality. In survival analysis, higher levels of log IL-6 were associated with increased CVD mortality (HR=1.70, 95%CI 1.54; 1.87). Standardising IL-6 by time of the day did not change the relationship (HR=1.71, 95%CI 1.55; 1.88). Also, standardising NT-ProBNP levels by time of the day did not alter the magnitude of the effect on CVD mortality (HR=1.92, 95%CI 1.81; 2.04). Finally, associations of t-PA levels with increased CVD mortality did not change substantially before (HR=1.74, 95%CI 1.45; 2.09) and after standardising (HR=1.77, 95%CI 1.47; 2.14) by time of the day.

Discussion

To our knowledge, this is the largest investigation of relationships between time of day and CVD risk factors in older men. After adjusting our analysis for potential confounding factors we demonstrated that some, but not all, CVD risk factors levels varied by time of day. In particular, NT-ProBNP and IL-6 increased linearly over the course of the day. Conversely, a decrease in t-PA was also observed; however, after accounting for fasting time the relationship with time of the day was strongly attenuated (therefore fasting time could partially explain the drop in t-PA levels observed in the afternoon vs morning). Our analyses showed that the contribution of time of the day to the overall variation of NT-ProBNP, IL-6, and t-PA was small and without clinical importance; we observed that time of day did not have a sufficiently strong effect to be taken into account when assessing the impact of IL-6,

For peer review only

NT-ProBNP, and t-PA on CVD mortality. Lastly, an association of time of day with other risk factors was not observed.

Literature on time of day variation in emerging CVD markers of inflammation and haemostasis in older adults is limited; to our knowledge this is the first time these findings have been reported in older adults. Findings from earlier studies of younger adults were fairly consistent with ours. For example a recent meta-analysis of several small studies which analysed IL-6 proposed a diurnal pattern, with overall IL-6 levels increased between 08:00 and 19:00 hours as in our study [16]. However, in two previous very small studies of twelve [17] and five [18] participants, IL-6 peaked in the night-time. It is possible that peaks in IL-6 levels may be associated with cognitive symptoms of depression [19] and daily activities, although in the BRHS population this has not yet been investigated. One previous study found that BRHS men were more active in the morning and in early afternoon [20] when the main activities were usually gardening, house works, shopping, or leisure walking. Whether IL-6 was implicated in this daily pattern remains uncertain and can potentially be explored in future studies.

Moreover, one previous study reported increased levels of NT-ProBNP over the course of day [21] as we observed in our study. A decrease in t-PA over the examination day was also reported in younger subjects (a 45-year-old UK population of 9377 men and women) [6]; however, t-PA did not vary by time of the day in a previous study large study of 1288 healthy 25 to 64-year-old men and women [22].

In comparison to our study, findings regarding CRP, Fibrinogen, D-dimer, vWF and cTnT reported in earlier studies of younger adults were similar: a few previous studies reported that they did not find an association of time of day with CRP [23], D-dimer [24], and vWF [25]. In one study, the variation in CRP, Fibrinogen, D-dimer, and vWF attributable to time of day was minimal [6]. Literature on cTnT is scarce: one small previous study of repeated measures in 7 participants with type 2 diabetes reported a decrease in cTnT between 8am and 8pm [26].

Although one previous study suggested that diurnal variations in CVD risk factors could be relevant for cardiovascular risk prediction [6], a prediction model like the one described in our

survival analysis was not performed. Our findings suggested the effect of time of the day (from 08:00 h to 19:00 h) is not relevant for the CVD risk assessment. With this sensitivity analysis we wanted to investigate time of day variations beyond simple descriptive diurnal patterns; to our knowledge this is the first time this finding has been reported.

Strengths and limitations

The BRHS cohort benefits from using a large scale population-based sample of free-living older men and this increases statistical power and precision of estimates. However, the BRHS comprises male participants, predominantly of white European ethnic origin, so findings may not be generalisable to women and non-white ethnic groups. The CVD risk factors measurements were carried out on one occasion over an extended period of the day (between 08:00 and 19:00 hours), offering only a partial understanding of the variations over the 24 hours [27 28]. Therefore, in this study the relationship of the CVD risk factors to time of day was explored using between-participant variation only. In future studies it may be advantageous to carry out repeated measurement of the risk factors over the 24 hours in order to investigate within-person circadian variations. However, with repeated measurements a possible and genuine diurnal variation may be disrupted and natural sleeping patterns altered (repeated measures are usually taken every 1-2 hours during the night) [29].

Implications

Variations of some CVD factors (in particular IL-6 and NT-ProBNP) over the course of the day were observed, suggesting the role of time of the day as potential confounder during the measurements. However, standardising these biological markers by time of day was not particularly relevant for the cardiovascular risk prediction. Also, other sensitivity analyses (stratified analysis and interaction tests) did not add relevant insights suggesting that time of day variations may be not important for clinical risk stratification in general. Further studies assessing both CVD risk factors levels and clinical outcomes (e.g. fatal or non-fatal CVD events) during 24h are required to demonstrate whether a rapid increase of IL-6 over the day may be relevant to the increased number of CVD events observed in early and late morning [30], and whether the increased levels of NT-ProBNP over the day are related to the afternoon peak in sudden death following heart failure [31].

For peer review only

Conclusions

Variations in time of day were associated with variations of some, but not all, CVD risk factors measured in older adults. The contribution of time of the day to the markers' overall variation was small and unlikely to affect the CVD risk prediction or clinical risk stratification.

AUTHORS CONTRIBUTIONS

CS processed the data, performed statistical analyses, drafted and revised the manuscript, and incorporated revisions of co-authors. PHW, SGW, BJ, and RM contributed to the design of the study and revised the manuscript. LL enrolled participants, and collected data. PHW, RM and SGW raised grant funding. All authors gave an intellectual contribution to the manuscript and approved the final version.

Acknowledgements

The BRHS is supported by a British Heart Foundation (BHF) programme grant (RG/13/16/30528). This research was supported by a BHF project grant (PG/13/41/30304) which supported CS. The funders had no role in the design and conduct of the study; collection, management, analysis, and interpretation of the data; preparation, review, or approval of the manuscript; and the decision to submit the manuscript for publication. The views expressed in this publication are those of the author(s) and not necessarily those of the BHF.

Conflict of interest statement

The authors report no relationships that could be construed as a conflict of interest

Data Sharing statement

The collection and management of data over the last 39 years of the BRHS has been made possible through grant funding from UK government agencies and charities. We welcome proposals for collaborative projects and data sharing. For general data sharing enquiries, please contact Lucy Lennon (l.lennon@ucl.ac.uk).

For peer review only

324 **Table 1** –Individual characteristics and risk factors levels in the British Regional Heart Study
325 (BRHS) men who attended the examinations in 1998-2000

Demographic and background characteristics	
Age (years), mean (Standard Deviation, SD)	68.7 (5.5)
Social class (manual)	
Manual, n (%)	2166 (51.1)
Non-Manual, n (%)	1966 (46.3)
Armed Forces, n (%)	112 (2.6)
Physical health	
Body Mass Index, mean (SD)	26.9 (3.7)
Prevalence of stroke/myocardial infarction, n (%)	153 (3.6)
Prevalence of heart failure, n (%)	390 (9.2)
Diabetes, n (%)	478 (11.2)
Behavioural factors	
Smoking	
Never, n (%)	1233 (29.1)
Ex-smokers, n (%)	2464 (58.0)
Smokers, n (%)	548 (12.9)
Alcohol consumption	
None, n (%)	431 (10.3)
Occasional/light, n (%) [1]	2949 (70.5)
Moderate/Heavy, n (%) [2]	779 (18.6)
Physical activity level	
Inactive, n (%)	471 (11.5)
Occasionally, n (%)	957 (23.4)
Light, n (%)	767 (18.7)
Moderate, n (%)	591 (14.4)
Moderate vigorous, n (%)	690 (16.8)
Vigorous, n (%)	621 (15.1)
CVD risk factor, geometric mean (SD) †	
CRP, mg/L	1.74 (3.03)
IL-6, pg/mL	2.46 (1.94)
Fibrinogen, g/L	3.19 (1.25)
t-PA, ng/mL	10.23 (1.50)
vWF, IU/dL	132.41 (1.40)
D-dimer, ng/mL	84.32 (2.32)
NT-ProBNP, pg/mL	101.50 (3.32)
cTnT, pg/mL	12.07 (1.64)

326 [1] >=1 and <=15 units per week (1 unit is approximately 1 drink, such as one glass of wine)
327 [2] >=16 units per week (1 unit is approximately 1 drink, such as one glass of wine)
328 † Abbreviations: C-Reactive Protein (CRP), Interleukin-6 (IL-6), Fibrinogen, tissue plasminogen activator (t-PA) ,
329 von Willebrand factor (vWF), fibrin D-dimer, N-terminal pro-brain natriuretic peptide (NT-ProBNP), and cardiac
330 Troponin T (cTnT)

Table 2 – Cross-sectional adjusted associations between time of day (fitted as continuous variable) and cardiovascular disease (CVD) risk factors measured in the British Regional Heart Study (BRHS) men (aged 60-79) attending the follow-up year 20 examination in 1998-2000. Associations are reported as percent difference in CVD risk factors levels per one hour of sampling over the examination day (08:00-19:00h). The statistically significant associations are marked in bold.

	Model 1: Age adjusted [1]		Model 2: Fully adjusted [2]		Model 3: Fully adjusted [2] excluding men with diabetes	
CVD risk factor [†]	Percent difference (95%CI) in the CVD risk factor levels per hour of sampling [3]	p-value	Percent difference (95%CI) in the CVD risk factor levels per hour of sampling [3]	p-value	Percent difference (95%CI) in the CVD risk factor levels per hour of sampling [4]	p-value
NT-ProBNP	**3.5 (2.0;5.0)**	**<0.001**	**3.3 (1.9; 4.8)**	**<0.001**	3.5 (2.0; 5.0)	**<0.001**
IL-6	**2.6 (1.7;3.4)**	**<0.001**	**2.6 (1.8; 3.4)**	**<0.001**	2.4 (1.6; 3.3)	**<0.001**
t-PA	**-3.3 (-3.8;-2.9)**	**<0.001**	**-3.3 (-3.7; -2.9)**	**<0.001**	-3.2 (-3.6; -2.7)	**<0.001**
Fibrinogen	-0.2 (-0.5;0.0)	0.088	-0.2 (-0.5; 0.1)	0.104	-0.2 (-0.5; 0.1)	0.149
cTnT	-0.4 (-0.9;0.2)	0.194	-0.4 (-1.0; 0.2)	0.174	-0.4 (-1.0; 0.2)	0.165
CRP	-1.0 (-2.3;0.4)	0.151	-0.9 (-2.2; 0.4)	0.175	-0.9 (-2.2; 0.5)	0.191
vWF	-0.2 (-0.6;0.2)	0.374	-0.2 (-0.6; 0.2)	0.380	-0.1 (-0.5; 0.3)	0.703
D-Dimer	-0.0 (-1.0;1.0)	0.929	-0.1 (-1.0; 0.9)	0.890	-0.1 (-1.2; 0.9)	0.801

[1] Model 1: Two level linear models (level 1 = person, level 2 = town of residence during the BRHS recruitment) adjusted for age. Model 1 used the same number of observations of Model 2 (complete case analysis).

[2] Model 1 additionally adjusted for social class, Body Mass Index, smoking status, alcohol consumption, physical activity, use of statin, and season. Associations with IL-6, t-PA, Fibrinogen, CRP, vWF, and D-Dimer were additionally adjusted for prevalence of stroke/MI, while association of time of the day with NT-ProBNP and cTnT models were additionally adjusted for prevalence of heart failure.

[3] Model 1 and Model 2 used the same number of observations: 3580 for NT-ProBNP, 3832 for IL-6, 3863 for t-PA, 3861 for Fibrinogen, 3827 for cTnT, 3838 for CRP, 3863 for vWF, 3859 for D-Dimer

[4] Model 3 number of observations: 3176 for NT-ProBNP, 3398 for IL-6, 3429 for t-PA, 3427 for Fibrinogen, 3398 for cTnT, 3405 for CRP, 3429 for vWF, 3425 for D-Dimer

[†] Abbreviations: C-Reactive Protein (CRP), Interleukin-6 (IL-6), Fibrinogen, tissue plasminogen activator (t-PA) , von Willebrand factor (vWF), fibrin D-dimer, N-terminal pro-brain natriuretic peptide (NT-ProBNP), and cardiac Troponin T (cTnT)

For peer review only

References

1. Pocock SJ, Ashby D, Shaper AG*, et al.* Diurnal variations in serum biochemical and haematological measurements. *Journal of clinical pathology*. 1989; 42: 172-9.
2. Emberson JR, Whincup PH, Walker M*, et al.* Biochemical measures in a population-based study: effect of fasting duration and time of day. *Annals of clinical biochemistry*. 2002; 39: 493-501.
3. Elliott WJ. Circadian variation in blood pressure: Implications for the elderly patient. *American Journal of Hypertension*. 1999; 12: 43S-9S.
4. IL6R Genetics Consortium and Emerging Risk Factors Collaboration. Interleukin-6 receptor pathways in coronary heart disease: a collaborative meta-analysis of 82 studies. *The Lancet*. 2012; 379: 1205-13.
5. Wannamethee SG, Shaper AG, Papacosta O*, et al.* Lung function and airway obstruction: associations with circulating markers of cardiac function and incident heart failure in older men-the British Regional Heart Study. *Thorax*. 2016; 71: 526-34.
6. Rudnicka AR, Rumley A, Lowe GD*, et al.* Diurnal, seasonal, and blood-processing patterns in levels of circulating fibrinogen, fibrin D-dimer, C-reactive protein, tissue plasminogen activator, and von Willebrand factor in a 45-year-old population. *Circulation*. 2007; 115: 996-1003.
7. Natriuretic Peptides Studies Collaboration. Natriuretic peptides and integrated risk assessment for cardiovascular disease: an individual-participant-data meta-analysis. *The lancet Diabetes & endocrinology*. 2016; 4: 840-9.
8. Shaper AG, Pocock SJ, Walker M*, et al.* British Regional Heart Study: cardiovascular risk factors in middle-aged men in 24 towns. *BMJ*. 1981; 283: 179-86.
9. Pocock SJ, Shaper AG, Cook DG*, et al.* British Regional Heart Study: geographic variations in cardiovascular mortality, and the role of water quality. *British Medical Journal*. 1980; 280: 1243-9.
10. Declaration of Helsinki - Ethical Principles for Medical Research Involving Human Subjects. *WMA Declaration of Helsinki*. WMA Declaration of HelsinkiOctober 2013.
11. Wannamethee G and Shaper AG. Physical activity and stroke in British middle aged men. *BMJ : British Medical Journal*. 1992; 304: 597-601.
12. Jefferis BJ, Sartini C, Ash S*, et al.* Validity of questionnaire-based assessment of sedentary behaviour and physical activity in a population-based cohort of older men; comparisons with objectively measured physical activity data. *Int J Behav Nutr Phys Act*. 2016; 13: 14.
13. Sartini C, Barry SJ, Wannamethee SG*, et al.* Effect of cold spells and their modifiers on cardiovascular disease events: Evidence from two prospective studies. *Int J Cardiol*. 2016; 218: 275-83.
14. Stolwijk AM, Straatman H and Zielhuis GA. Studying seasonality by using sine and cosine functions in regression analysis. *Journal of Epidemiology and Community Health*. 1999; 53: 235-8.
15. Wannamethee SG, Whincup PH, Lennon L*, et al.* Associations Between Fibrin D-Dimer, Markers of Inflammation, Incident Self-Reported Mobility Limitation, and All-Cause Mortality in Older Men. *Journal of the American Geriatrics Society*. 2014; 62: 2357-62.
16. Nilsonne G, Lekander M, Åkerstedt T*, et al.* Diurnal variation of circulating interleukin-6 in humans: a meta-analysis. *bioRxiv*. 2016.

For peer review only

17. Gudewill S, Pollmacher T, Vedder H, *et al.* Nocturnal plasma levels of cytokines in healthy men. *European archives of psychiatry and clinical neuroscience*. 1992; 242: 53-6.
18. Bauer J, Hohagen F, Ebert T, *et al.* Interleukin-6 serum levels in healthy persons correspond to the sleep-wake cycle. *The Clinical investigator*. 1994; 72: 315-.
19. Gimeno D, Kivimaki M, Brunner EJ, *et al.* Associations of C-reactive protein and interleukin-6 with cognitive symptoms of depression: 12-year follow-up of the Whitehall II study. *Psychological medicine*. 2009; 39: 413-23.
20. Sartini C, Wannamethee SG, Iliffe S, *et al.* Diurnal patterns of objectively measured physical activity and sedentary behaviour in older men. *BMC Public Health*. 2015; 15: 609.
21. Goetze JP, Jorgensen HL, Sennels HP, *et al.* Diurnal plasma concentrations of natriuretic propeptides in healthy young males. *Clinical chemistry*. 2012; 58: 789-92.
22. Eliasson M, Evrin PE, Lundblad D, *et al.* Influence of gender, age and sampling time on plasma fibrinolytic variables and fibrinogen. *Fibrinolysis*. 1993; 7: 316-23.
23. Meier-Ewert HK, Ridker PM, Rifai N, *et al.* Absence of diurnal variation of C-reactive protein concentrations in healthy human subjects. *Clinical chemistry*. 2001; 47: 426-30.
24. Jafri SM, VanRollins M, Ozawa T, *et al.* Circadian variation in platelet function in healthy volunteers. *Am J Cardiol*. 1992; 69: 951-4.
25. Bridges AB, McLaren M, Scott NA, *et al.* Circadian variation of tissue plasminogen activator and its inhibitor, von Willebrand factor antigen, and prostacyclin stimulating factor in men with ischaemic heart disease. *British Heart Journal*. 1993; 69: 121-4.
26. Klinkenberg LJ, van Dijk JW, Tan FE, *et al.* Circulating cardiac troponin T exhibits a diurnal rhythm. *Journal of the American College of Cardiology*. 2014; 63: 1788-95.
27. Bremner WF, Sothern RB, Kanabrocki EL, *et al.* Relation between circadian patterns in levels of circulating lipoprotein(a), fibrinogen, platelets, and related lipid variables in men. *American heart journal*. 2000; 139: 164-73.
28. Kristal-Boneh E, Harari G and Green MS. Seasonal Change in 24-Hour Blood Pressure and Heart Rate Is Greater Among Smokers Than Nonsmokers. *Hypertension*. 1997; 30: 436-41.
29. Vgontzas AN, Zoumakis M, Bixler EO, *et al.* Impaired nighttime sleep in healthy old versus young adults is associated with elevated plasma interleukin-6 and cortisol levels: physiologic and therapeutic implications. *J Clin Endocrinol Metab*. 2003; 88: 2087-95.
30. Elliott WJ. Circadian variation in the timing of stroke onset: a meta-analysis. *Stroke; a journal of cerebral circulation*. 1998; 29: 992-6.
31. Carson PA, O'Connor CM, Miller AB, *et al.* Circadian rhythm and sudden death in heart failureResults from prospective randomized amlodipine survival trial. *Journal of the American College of Cardiology*. 2000; 36: 541-6.

For peer review only

Figure 1 – Unadjsted geometric means (95% CI) by time of the day [1] for C-Reactive Protein (CRP), Interleukin-6 (IL-6), Fibrinogen, tissue plasminogen activator (t-PA) , von Willebrand factor (vWF), fibrin D-dimer, N-terminal pro-brain natriuretic peptide (NT-ProBNP), and cardiac Troponin T (cTnT) measured on one occasion in BRHS men aged 60-79 during the years 1998-2000.

[1] Total number of men examined per hour was 33(0.7%) at 08:00-08:59, 363(8.5%) at 8:00-9:59h, 699(16%) at 10:00-10:59h, 771(18%) at 11:00-11:59h, 591(14%) at 12:00-12:59h, 99(2%) at 13:00-13:59h, 306(7%) at 14:00-14:59), 560(13%) at 15:00-15:59h, 566(13%) at 16:00-16:59, 260(6%) at 17:00-17:59, and 3(<0.1%) at 18:00-18:59

For peer review only

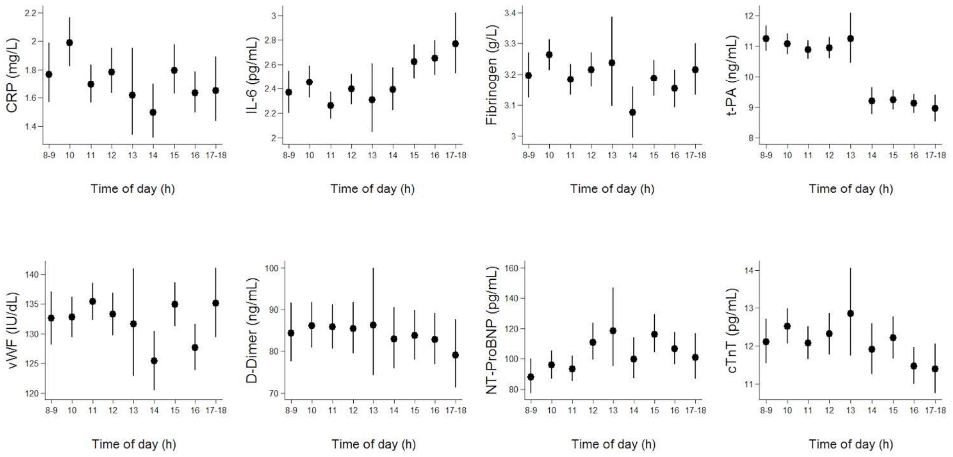

Figure 1 – Unadjsted geometric means (95% CI) by time of the day

186x99mm (300 x 300 DPI)

review only

STROBE Statement—Checklist of items that should be included in reports of ***cross-sectional studies***

	Item No	Recommendation	
**Title and abstract**	1	(*a*) Indicate the study's design with a commonly used term in the title or the abstract	Abstract
		(*b*) Provide in the abstract an informative and balanced summary of what was done and what was found	Abstract
**Introduction**			
Background/rationale	2	Explain the scientific background and rationale for the investigation being reported	Introduction, lines 54-62
Objectives	3	State specific objectives, including any prespecified hypotheses	Introduction, lines 54-56 and 60-62
**Methods**			
Study design	4	Present key elements of study design early in the paper	Methods, line 66-72
Setting	5	Describe the setting, locations, and relevant dates, including periods of recruitment, exposure, follow-up, and data collection	Methods, lines 75-84
Participants	6	(*a*) Give the eligibility criteria, and the sources and methods of selection of participants	Methods, lines 66-72 snd 75-84
Variables	7	Clearly define all outcomes, exposures, predictors, potential confounders, and effect modifiers. Give diagnostic criteria, if applicable	Methods , lines 97-123
Data sources/ measurement	8*	For each variable of interest, give sources of data and details of methods of assessment (measurement). Describe comparability of assessment methods if there is more than one group	Methods , lines 75-123
Bias	9	Describe any efforts to address potential sources of bias	
Study size	10	Explain how the study size was arrived at 4252	Results, lines 75-78
Quantitative variables	11	Explain how quantitative variables were handled in the analyses. If applicable, describe which groupings were chosen and why	Methods , lines 91-114
Statistical methods	12	(*a*) Describe all statistical methods, including those used to control for confounding	Methods , lines 125-159
		(*b*) Describe any methods used to examine subgroups and interactions	Methods , lines 152 and 156-158
		(*c*) Explain how missing data were addressed	
		(*d*) If applicable, describe analytical methods taking account of sampling strategy	NA
		(*e*) Describe any sensitivity analyses	Methods , lines 151-159
**Results**			
Participants	13*	(a) Report numbers of individuals at each stage of study—eg numbers potentially eligible, examined for eligibility, confirmed eligible, included in the study, completing follow-up, and analysed	Methods, , lines 66-68 and 91-95
		(b) Give reasons for non-participation at each stage	Methods, lines 66-76
		(c) Consider use of a flow diagram	Not necessary for this study, see Methods, lines 66-68 and 91-95

For peer review only - http://bmjopen.bmj.com/site/about/guidelines.xhtml

Descriptive data	14*	(a) Give characteristics of study participants (eg demographic, clinical, social) and information on exposures and potential confounders	Table 1
		(b) Indicate number of participants with missing data for each variable of interest	Table 1
Outcome data	15*	Report numbers of outcome events or summary measures	Table 1, Figure 1
Main results	16	(*a*) Give unadjusted estimates and, if applicable, confounder-adjusted estimates and their precision (eg, 95% confidence interval). Make clear which confounders were adjusted for and why they were included	Table 2
		(*b*) Report category boundaries when continuous variables were categorized	NA
		(*c*) If relevant, consider translating estimates of relative risk into absolute risk for a meaningful time period	NA
Other analyses	17	Report other analyses done—eg analyses of subgroups and interactions, and sensitivity analyses	Results, lines 187-195
**Discussion**			
Key results	18	Summarise key results with reference to study objectives	Results, lines 170-181 Discussion, lines 224-235
Limitations	19	Discuss limitations of the study, taking into account sources of potential bias or imprecision. Discuss both direction and magnitude of any potential bias	Discussion, lines lines 272-284
Interpretation	20	Give a cautious overall interpretation of results considering objectives, limitations, multiplicity of analyses, results from similar studies, and other relevant evidence	Discussion, lines 230-234 and 272 - 284
Generalisability	21	Discuss the generalisability (external validity) of the study results	Discussion, lines 287-289
**Other information**			
Funding	22	Give the source of funding and the role of the funders for the present study and, if applicable, for the original study on which the present article is based	Acknowledgements, lines 287-302

*Give information separately for exposed and unexposed groups.

Note: An Explanation and Elaboration article discusses each checklist item and gives methodological background and published examples of transparent reporting. The STROBE checklist is best used in conjunction with this article (freely available on the Web sites of PLoS Medicine at http://www.plosmedicine.org/, Annals of Internal Medicine at http://www.annals.org/, and Epidemiology at http://www.epidem.com/). Information on the STROBE Initiative is available at www.strobe-statement.org.

For peer review only

For peer review only - http://bmjopen.bmj.com/site/about/guidelines.xhtml

BMJ Open

Associations of time of day with cardiovascular disease risk factors measured in older men: results from the British Regional Heart Study

Journal:	*BMJ Open*
Manuscript ID	bmjopen-2017-018264.R2
Article Type:	Research
Date Submitted by the Author:	25-Aug-2017
Complete List of Authors:	Sartini, Claudio; University College of London, Primary Care and Population Health<br>Whincup, Peter; St Georges, University of London, Population Health Research Institute<br>Wannamethee, Goya; University College London<br>Jefferis, Barbara ; University College London<br>Lennon, Lucy; UCL, Department of Primary Care and Population Health<br>Lowe, Gordon; University of Glasgow, Institute of Cardiovascular and Medical Sciences<br>Welsh, Paul; University of Glasgow, Institute of Cardiovascular and Medical Sciences<br>Sattar, Naveed; University of Glasgow, BHF centre<br>Morris, Richard; University of Bristol, School of Social & Community Medicine
**Primary Subject Heading**:	Epidemiology
Secondary Subject Heading:	Public health
Keywords:	biological markers, older adults, cardiovascular disease, diurnal variations

SCHOLARONE™
Manuscripts

Title: Associations of time of day with cardiovascular disease risk factors measured in older men: results from the British Regional Heart Study

Claudio Sartini, MSc [1 *], Peter H Whincup, PhD [2], S Goya Wannamethee, PhD [1], Barbara J Jefferis, PhD [1], Lucy Lennon, MSc [1], Gordon DO Lowe, PhD [3], Paul Welsh, PhD [3], Naveed Sattar, PhD [3], and Richard W Morris, PhD [4]

[1] Department of Primary Care & Population Health, University College London, Rowland Hill Street, NW3 2PF, London. UK

[2] Population Health Research Institute, St George's University of London, Cranmer Terrace London SW17 0RE, UK.

[3] Institute of Cardiovascular and Medical Sciences, University of Glasgow, Glasgow G12 8TA UK

[4] School of Social & Community Medicine, University of Bristol, Canynge Hall, 39 Whatley Rd, Bristol BS8 2PS, UK.

[*] Corresponding Author

E-mail: c.sartini@ucl.ac.uk;

Manuscript number of words: 4847 words (all included: title page, abstract, manuscript, all tables and references).

For peer review only

Abstract (250 words, now 248)

Objective: We estimated associations of time of day with cardiovascular disease (CVD) risk factors measured in older men.

Methods: CVD risk factors (markers of inflammation and haemostasis, and cardiac markers) were measured on one occasion between 08:00-19:00 hours in 4252 men aged 60-79 years from the British Regional Heart Study. Linear models were used to estimate associations between time of day and risk factors. When an association was found, we examined whether the relationship between risk factors and cardiovascular mortality was affected by the adjustment for time of day using survival analyses.

Results: N-terminal pro-brain natriuretic peptide (NT-ProBNP) levels increased by 3.3% per hour [95% Confidence interval (CI) 1.9; 4.8], Interleukin-6 (IL-6) increased by 2.6% per hour (95% CI 1.8; 3.4), while Tissue plasminogen activator (t-PA) decreased by 3.3% per hour (95% CI 3.7; 2.9); these associations were unaffected by adjustment for possible confounding factors. The percentages of variation in these risk factors attributable to time of day were less than 2%. In survival analyses, the association of IL-6, NT-ProBNP, and t-PA with cardiovascular mortality was not affected by the adjustment for time of day. C-Reactive Protein, Fibrinogen, D-Dimer, von Willebrand Factor, and cardiac Troponin T showed no associations with time of day.

Conclusions: In older men, markers of inflammation (IL-6), haemostasis (t-PA), and a cardiac marker (NT-ProBNP) varied by time of day. The contribution of time of day to variations in these markers was small, and did not appear to be relevant for the CVD risk prediction.

For peer review only

Background

Previous studies have reported time of day variation in both established and emerging cardiovascular disease (CVD) risk factors in middle aged adults, such as blood pressure, lipids and some well-established inflammatory and haemostatic factors (e.g. white blood cell, red blood cell, and platelets counts) [1-3]. However, the extent to which emerging CVD risk factors such as Interleukin-6, a marker of inflammation causally associated with CHD in a recent study [4], and N-terminal pro-brain natriuretic peptide, a marker of heart failure [5] vary by time of day have been less studied. Moreover, very little is known on time of day variations in other emerging risk factors prospectively associated with CVD (e.g. t-PA, D-Dimer, von Willebrand factor, and Cardiac Troponin T), although their causal association with CVD remain debated or not yet tested.

We would expect that time of day variations in some emerging CVD risk factors measured in older adults may occur, consistent with findings in younger populations [6]. However, in older adults the degree of difference attributable to time of day has not been yet estimated; establishing its importance and its effects on prediction of CVD risk is important given the potentially wider use of N-terminal pro-brain natriuretic peptide in risk stratification (as shown in a recent major meta-analysis in the general population [7]), and potential causal link between IL-6 and cardiovascular disease [4]. Therefore, the aim of this study was to investigate how emerging CVD risk factors, including markers of inflammation, haemostasis and myocardial function, varied by time of day in older British men.

Methods

Participants

The British Regional Heart Study (BRHS) is a prospective cohort study of cardiovascular disease involving 7735 middle aged men (40-59 years) selected in 1978-80 from the age-sex registers of one local primary care centre in 24 British towns [8]. The 24 towns were selected to represent the variation in cardiovascular disease across the UK [9]. The National Research Ethics Service (NRES) Committee for London provided ethical approval. Participants provided informed written consent to the investigation, which was performed in accordance with the Declaration of Helsinki [10].

For peer review only

Follow-up examination

In 1998-2000, an average of 20 years after the initial recruitment, 4252 surviving participants (77% response rate) aged 60-79 years who were resident in the UK attended a physical examination during which nurses took a fasting blood sample on one occasion for each participant. The men were asked to fast for a minimum of 6 hours, during which they were instructed to drink only water, as previously reported [2]. The blood samples were collected between 08:00 h and 19:00 h and then assayed for a range of biochemical and haematological markers. Participants' appointment times were non-systematically allocated. They were offered the opportunity to contact the BRHS team and change the time of examination, if unable to attend; a small proportion of participants did so.

The participants were also asked to complete a questionnaire which included questions on other established CVD risk factors, such as age, social class, smoking habits, alcohol consumption, and physical activity. Specifically, physical activity levels were self-reported [11] and recently validated using accelerometers [12]. Incident CVD, including non-fatal stroke and non-fatal MI were recorded: their definitions have been reported elsewhere [13]. Men were also asked whether a doctor had ever told them that they had heart failure [5]. The number of blood samples collected and included in the analyses differ according to the risk factor measurements (the number of observations varied from 3580 for N-terminal Pro-Brain Natriuretic Peptide to 3863 for von Willebrand Factor in complete case analyses including all covariates of interest).

CVD Risk factors

Circulating levels of markers of inflammation (C-reactive protein [CRP], Interleukin 6 [IL-6], Fibrinogen), cardiac markers (N-terminal pro-brain natriuretic peptide [NT-ProBNP], cardiac Troponin T [cTnT], and markers of haemostasis (tissue plasminogen activator [t-PA] antigen, fibrin D-dimer, von Willebrand factor [vWF]) were measured.

D-dimer and t-PA levels were measured using an enzyme-linked immunosorbent assay (ELISA; Biopool AB, Umeå, Sweden), as was VWF antigen (Dako, High Wycombe, UK). C-reactive protein was assayed using ultrasensitive nephelometry (Dade Behring, Milton Keynes, UK). IL-6 was assayed using a high-sensitivity ELISA (R & D Sys-tems, Oxford, UK).

For peer review only

107 Fibrinogen was assayed using an auto-mated Clauss assay in a coagulometer (MDA-180, Organon Teknika, Cambridge, UK). NT-proBNP and hsTnT were measured in plasma samples on an automated clinically validated immunoassay analyzer (e411, Roche Diagnostics, Burgess Hill, United Kingdom) using the manufacturers' calibrators and quality control reagents. Intra- and inter-assay Coefficient of Variations (CVs) were, respectively: 4.1% and 6.6% for t-PA; 3.2% and 4.2% for vWF; 4.7% and 5.2% for D-dimer; 4.7% and 8.3% for CRP; 7.5% and 8.9% for IL-6; 2.6% and 3.7% for Fibrinogen, and 4.4% and 7.7% for NT-ProBNP and cTnT.

The samples were centrifuged and separated on the morning or afternoon of collection and stored on site at -20°C until they were transferred to a central freezer storage location at -70°C within 2 weeks of sample collection. Samples were then transferred on dry ice to a single central laboratory and were thawed immediately before analysis. Plasma samples were used for all the analyses reported here. The original sample collection took place between January 1998 and March 2000. Most of the analyses described here were carried out during 2000, after a maximum of 3 years storage; NT-ProBNP and cTNT were analysed in 2009.

Statistical methods

Firstly, the distributions of the outcomes were examined; the outcomes were log-transformed as the distributions were positively skewed. Therefore, analysis was carried out on their log-transformed values throughout. Unadjusted geometric means and 95% Confidence Intervals [CI] of the outcomes were plotted against hour of the day.

Adjusted associations between time of day and the outcomes

Associations between time of day (fitted as a continuous variable, range 8-18) and the outcomes were examined using linear multilevel random intercept models (level 1 = individual, level 2 = town of residence). The results can be interpreted as between-person variations over the course of the examination day; the estimates from the linear model were reported as the difference in the outcome levels per hour of sampling over the examination day. As the outcomes were log-transformed, the results were reported as percent difference in the outcome geometric mean per hour of sampling. All models were

For peer review only

initially adjusted for age only. Next, the models were adjusted for age and other possible confounding factors: social class, BMI, previous stroke or myocardial infarction (MI), physical activity, smoking status, alcohol consumption, use of statin, and a seasonal term (fitted using a cosinor function, as in previous studies) [14]. As NT-ProBNP and cTnT are principally markers of heart failure, the association with time of day was adjusted for previous heart failure.

When the association of time of the day with the outcomes was found to be statistically significant, the proportion of variance associated with time of the day was estimated using partial R-squared.

Sensitivity analyses

Six sensitivity analyses were performed: (i) all models were additionally adjusted for fasting time and diabetes; (ii) all models were carried out excluding men with diabetes, (iii) interactions were fitted to test whether the time of day associations were modified by age (fitted as continuous variable); (iv) as NT-ProBNP and cTnT were acknowledged as specific cardiac markers [5], interactions were fitted to test whether the time of day associations were modified by previous heart failure (yes/no); (v) to explore the potential of undiagnosed heart failure or cardiac damage influencing findings for NT-ProBNP and cTnT, we repeated regression models after excluding men with NTproBNP > 400 pg/ml; (vi) a quadratic term for time of day was added to the models in order to check for non-linearity

As IL-6 has been causally associated with cardiovascular risk [4], and prospectively associated with CVD mortality in the BRHS sample used here [15], we investigated the relevance of time of day to the cardiovascular risk prediction by performing two survival analyses: in the first analysis we used Cox models where unadjusted log IL-6 was used as the predictor and CVD mortality as the clinical outcome; then, we repeated the same analysis using log IL-6 standardised by the time of day rather than unadjusted log IL-6. For completeness of information, we repeated this sensitivity analysis for NT-ProBNP and t-PA.

Results

The characteristics of the study participants (mean age 68.7 years, standard deviation (SD) =

For peer review only

5.5) are reported in Table 1. The associations between time of day (by hour) and risk factors are shown in Figure 1. Evidence of an increase over the course of the day was particularly noticeable for IL-6, and for NT-ProBNP (Figure 1). Also, levels of t-PA were lower in the afternoon in comparison with morning, while variations by time of day for other risk factors were not clearly observable from the plots (Figure 1). The results of corresponding linear regression analyses are shown in Table 2: statistically significant associations between time of the day and some outcomes were found (Table 2): over the course of the examination day NT-ProBNP levels increased by 3.3% per hour (95% CI 1.9; 4.8%), IL-6 increased by 2.6% per hour (95% CI 1.8; 3.4%). Conversely, t-PA decreased by 3.3% per hour (95% CI 3.7; 2.9%). The proportion of variance associated with time of the day from the fully adjusted models was 0.5%, 1%, and 2% for NT-ProBNP, IL-6, and t-PA respectively.

C-Reactive Protein, Fibrinogen, D-Dimer, von Willebrand Factor, and cardiac Troponin T showed no consistent associations with time of day (Table 2).

Sensitivity analyses

Overall, we found that fasting time did not alter the magnitude of associations between time of the day and the outcomes reported in Table 2. Only the association between time of the day and t-PA was strongly attenuated after accounting for fasting time (fitted as continuous variable): the decrease in t-PA levels was -3.3% (95%CI -3.7; -2.9) per hour before the adjustment (Table 2) and -1.4% (95%CI -2.2; -0.1) after the adjustment for fasting. An additional adjustment for diabetes status did not alter the magnitude of the association between hour of the day and the outcomes. We also performed the analysis excluding men with diabetes completely (Table 2 – Model 3), but the association between time of day and the outcomes did not substantially change.

For all outcomes, we also did not find evidence for an interaction between of time of day with age (results not shown).

In stratified analysis, NT-ProBNP levels increased by 3.4% (95% CI 1.9; 4.8%, $p<0.001$) per hour in older men without heart failure. Although men who previously had heart failure had increased NT-ProBNP levels, there was no evidence for an interaction between previous

For peer review only

heart failure with time of the day (p=0.954). After excluding 466 men with NTproBNP levels >400pg/ml (12% of the sample), associations between time of the day measures and NTproBNP remained statistically significant and slightly increased in magnitude (3.9% [95% CI 2.7; 5.1%], p<0.001). As reported in the main analysis, no significant associations were found between time of the day and cTnT in stratified analysis.

When adding a quadratic term to the model, we found a significant improvement in model fit for IL-6 only (p=0.030 for the time of day squared term). The association of time of day with IL-6 appeared to be slightly J-shaped, with a linear increase starting from 11:00 until 19:00 hours.

We examined whether adjustment for hour of day affected the associations between risk factors and CVD mortality. In survival analysis, higher levels of log IL-6 were associated with increased CVD mortality (HR=1.70, 95%CI 1.54; 1.87). Standardising IL-6 by time of the day did not change the relationship (HR=1.71, 95%CI 1.55; 1.88). Also, standardising NT-ProBNP levels by time of the day did not alter the magnitude of the effect on CVD mortality (HR=1.92, 95%CI 1.81; 2.04). Finally, associations of t-PA levels with increased CVD mortality did not change substantially before (HR=1.74, 95%CI 1.45; 2.09) and after standardising (HR=1.77, 95%CI 1.47; 2.14) by time of the day.

Discussion

To our knowledge, this is the largest investigation of relationships between time of day and CVD risk factors in older men. After adjusting our analysis for potential confounding factors we demonstrated that some, but not all, CVD risk factors levels varied by time of day. In particular, NT-ProBNP and IL-6 increased linearly over the course of the day. Conversely, a decrease in t-PA was also observed; however, after accounting for fasting time the relationship with time of the day was strongly attenuated (therefore fasting time could partially explain the drop in t-PA levels observed in the afternoon vs morning). Our analyses showed that the contribution of time of the day to the overall variation of NT-ProBNP, IL-6, and t-PA was small and without clinical importance; we observed that time of day did not have a sufficiently strong effect to be taken into account when assessing the impact of IL-6,

For peer review only

NT-ProBNP, and t-PA on CVD mortality. Lastly, an association of time of day with other risk factors was not observed.

Literature on time of day variation in emerging CVD markers of inflammation and haemostasis in older adults is limited; to our knowledge this is the first time these findings have been reported in older adults. Findings from earlier studies of younger adults were fairly consistent with ours. For example a recent meta-analysis of several small studies which analysed IL-6 proposed a diurnal pattern, with overall IL-6 levels increased between 08:00 and 19:00 hours as in our study [16]. However, in two previous very small studies of twelve [17] and five [18] participants, IL-6 peaked in the night-time. It is possible that peaks in IL-6 levels may be associated with cognitive symptoms of depression [19] and daily activities, although in the BRHS population this has not yet been investigated. One previous study found that BRHS men were more active in the morning and in early afternoon [20] when the main activities were usually gardening, house works, shopping, or leisure walking. Whether IL-6 was implicated in this daily pattern remains uncertain and can potentially be explored in future studies.

Moreover, one previous study reported increased levels of NT-ProBNP over the course of day [21] as we observed in our study. A decrease in t-PA over the examination day was also reported in younger subjects (a 45-year-old UK population of 9377 men and women) [6]; however, t-PA did not vary by time of the day in a previous study large study of 1288 healthy 25 to 64-year-old men and women [22].

In comparison to our study, findings regarding CRP, Fibrinogen, D-dimer, vWF and cTnT reported in earlier studies of younger adults were similar: a few previous studies reported that they did not find an association of time of day with CRP [23], D-dimer [24], and vWF [25]. In one study, the variation in CRP, Fibrinogen, D-dimer, and vWF attributable to time of day was minimal [6]. Literature on cTnT is scarce: one small previous study of repeated measures in 7 participants with type 2 diabetes reported a decrease in cTnT between 8am and 8pm [26].

Although one previous study suggested that diurnal variations in CVD risk factors could be relevant for cardiovascular risk prediction [6], a prediction model like the one described in our

For peer review only

survival analysis was not performed. Our findings suggested the effect of time of the day (from 08:00 h to 19:00 h) is not relevant for the CVD risk assessment. With this sensitivity analysis we wanted to investigate time of day variations beyond simple descriptive diurnal patterns; to our knowledge this is the first time this finding has been reported.

Strengths and limitations

The BRHS cohort benefits from using a large scale population-based sample of free-living older men and this increases statistical power and precision of estimates. However, the BRHS comprises male participants, predominantly of white European ethnic origin, so findings may not be generalisable to women and non-white ethnic groups. The CVD risk factors measurements were carried out on one occasion over an extended period of the day (between 08:00 and 19:00 hours), offering only a partial understanding of the variations over the 24 hours [27 28]. Therefore, in this study the relationship of the CVD risk factors to time of day was explored using between-participant variation only. In future studies it may be advantageous to carry out repeated measurement of the risk factors over the 24 hours in order to investigate within-person circadian variations. However, with repeated measurements a possible and genuine diurnal variation may be disrupted and natural sleeping patterns altered (repeated measures are usually taken every 1-2 hours during the night) [29].

Implications

Variations of some CVD factors (in particular IL-6 and NT-ProBNP) over the course of the day were observed, suggesting the role of time of the day as potential confounder during the measurements. However, standardising these biological markers by time of day was not particularly relevant for the cardiovascular risk prediction. Also, other sensitivity analyses (stratified analysis and interaction tests) did not add relevant insights suggesting that time of day variations may be not important for clinical risk stratification in general. Further studies assessing both CVD risk factors levels and clinical outcomes (e.g. fatal or non-fatal CVD events) during 24h are required to demonstrate whether a rapid increase of IL-6 over the day may be relevant to the increased number of CVD events observed in early and late morning [30], and whether the increased levels of NT-ProBNP over the day are related to the afternoon peak in sudden death following heart failure [31].

For peer review only

Conclusions

Variations in time of day were associated with variations of some, but not all, CVD risk factors measured in older adults. The contribution of time of the day to the markers' overall variation was small and unlikely to affect the CVD risk prediction or clinical risk stratification.

AUTHORS CONTRIBUTIONS

CS processed the data, performed statistical analyses, drafted and revised the manuscript, and incorporated revisions of co-authors. PHW, SGW, BJ, and RM contributed to the design of the study and revised the manuscript. LL enrolled participants, and collected data. PHW, RM and SGW raised grant funding. All authors gave an intellectual contribution to the manuscript and approved the final version.

Acknowledgements

The BRHS is supported by a British Heart Foundation (BHF) programme grant (RG/13/16/30528). This research was supported by a BHF project grant (PG/13/41/30304) which supported CS. The funders had no role in the design and conduct of the study; collection, management, analysis, and interpretation of the data; preparation, review, or approval of the manuscript; and the decision to submit the manuscript for publication. The views expressed in this publication are those of the author(s) and not necessarily those of the BHF.

Conflict of interest statement

The authors report no relationships that could be construed as a conflict of interest

Data Sharing statement

The collection and management of data over the last 39 years of the BRHS has been made possible through grant funding from UK government agencies and charities. We welcome proposals for collaborative projects and data sharing. For general data sharing enquiries, please contact Lucy Lennon (l.lennon@ucl.ac.uk).

For peer review only

Table 1 –Individual characteristics and risk factors levels in the British Regional Heart Study (BRHS) men who attended the examinations in 1998-2000

Demographic and background characteristics	
Age (years), mean (Standard Deviation, SD)	68.7 (5.5)
Social class (manual)	
Manual, n (%)	2166 (51.1)
Non-Manual, n (%)	1966 (46.3)
Armed Forces, n (%)	112 (2.6)
Physical health	
Body Mass Index, mean (SD)	26.9 (3.7)
Prevalence of stroke/myocardial infarction, n (%)	153 (3.6)
Prevalence of heart failure, n (%)	390 (9.2)
Diabetes, n (%)	478 (11.2)
Behavioural factors	
Smoking	
Never, n (%)	1233 (29.1)
Ex-smokers, n (%)	2464 (58.0)
Smokers, n (%)	548 (12.9)
Alcohol consumption	
None, n (%)	431 (10.3)
Occasional/light, n (%) [1]	2949 (70.5)
Moderate/Heavy, n (%) [2]	779 (18.6)
Physical activity level	
Inactive, n (%)	471 (11.5)
Occasionally, n (%)	957 (23.4)
Light, n (%)	767 (18.7)
Moderate, n (%)	591 (14.4)
Moderate vigorous, n (%)	690 (16.8)
Vigorous, n (%)	621 (15.1)
CVD risk factor, geometric mean (SD) †	
CRP, mg/L	1.74 (3.03)
IL-6, pg/mL	2.46 (1.94)
Fibrinogen, g/L	3.19 (1.25)
t-PA, ng/mL	10.23 (1.50)
vWF, IU/dL	132.41 (1.40)
D-dimer, ng/mL	84.32 (2.32)
NT-ProBNP, pg/mL	101.50 (3.32)
cTnT, pg/mL	12.07 (1.64)

[1] >=1 and <=15 units per week (1 unit is approximately 1 drink, such as one glass of wine)

[2] >=16 units per week (1 unit is approximately 1 drink, such as one glass of wine)

† Abbreviations: C-Reactive Protein (CRP), Interleukin-6 (IL-6), Fibrinogen, tissue plasminogen activator (t-PA) , von Willebrand factor (vWF), fibrin D-dimer, N-terminal pro-brain natriuretic peptide (NT-ProBNP), and cardiac Troponin T (cTnT)

For peer review only

Table 2 – Cross-sectional adjusted associations between time of day (fitted as continuous variable) and cardiovascular disease (CVD) risk factors measured in the British Regional Heart Study (BRHS) men (aged 60-79) attending the follow-up year 20 examination in 1998-2000. Associations are reported as percent difference in CVD risk factors levels per one hour of sampling over the examination day (08:00-19:00h). The statistically significant associations are marked in bold.

	Model 1: Age adjusted [1]		Model 2: Fully adjusted [2]		Model 3: Fully adjusted [2] excluding men with diabetes	
CVD risk factor [†]	Percent difference (95%CI) in the CVD risk factor levels per hour of sampling [3]	p-value	Percent difference (95%CI) in the CVD risk factor levels per hour of sampling [3]	p-value	Percent difference (95%CI) in the CVD risk factor levels per hour of sampling [4]	p-value
NT-ProBNP	**3.5 (2.0;5.0)**	**<0.001**	**3.3 (1.9; 4.8)**	**<0.001**	3.5 (2.0; 5.0)	**<0.001**
IL-6	**2.6 (1.7;3.4)**	**<0.001**	**2.6 (1.8; 3.4)**	**<0.001**	2.4 (1.6; 3.3)	**<0.001**
t-PA	**-3.3 (-3.8;-2.9)**	**<0.001**	**-3.3 (-3.7; -2.9)**	**<0.001**	-3.2 (-3.6; -2.7)	**<0.001**
Fibrinogen	-0.2 (-0.5;0.0)	0.088	-0.2 (-0.5; 0.1)	0.104	-0.2 (-0.5; 0.1)	0.149
cTnT	-0.4 (-0.9;0.2)	0.194	-0.4 (-1.0; 0.2)	0.174	-0.4 (-1.0; 0.2)	0.165
CRP	-1.0 (-2.3;0.4)	0.151	-0.9 (-2.2; 0.4)	0.175	-0.9 (-2.2; 0.5)	0.191
vWF	-0.2 (-0.6;0.2)	0.374	-0.2 (-0.6; 0.2)	0.380	-0.1 (-0.5; 0.3)	0.703
D-Dimer	-0.0 (-1.0;1.0)	0.929	-0.1 (-1.0; 0.9)	0.890	-0.1 (-1.2; 0.9)	0.801

[1] Model 1: Two level linear models (level 1 = person, level 2 = town of residence during the BRHS recruitment) adjusted for age. Model 1 used the same number of observations of Model 2 (complete case analysis).

[2] Model 1 additionally adjusted for social class, Body Mass Index, smoking status, alcohol consumption, physical activity, use of statin, and season. Associations with IL-6, t-PA, Fibrinogen, CRP, vWF, and D-Dimer were additionally adjusted for prevalence of stroke/MI, while association of time of the day with NT-ProBNP and cTnT models were additionally adjusted for prevalence of heart failure.

[3] Model 1 and Model 2 used the same number of observations: 3580 for NT-ProBNP, 3832 for IL-6, 3863 for t-PA, 3861 for Fibrinogen, 3827 for cTnT, 3838 for CRP, 3863 for vWF, 3859 for D-Dimer

[4] Model 3 number of observations: 3176 for NT-ProBNP, 3398 for IL-6, 3429 for t-PA, 3427 for Fibrinogen, 3398 for cTnT, 3405 for CRP, 3429 for vWF, 3425 for D-Dimer

[†] Abbreviations: C-Reactive Protein (CRP), Interleukin-6 (IL-6), Fibrinogen, tissue plasminogen activator (t-PA) , von Willebrand factor (vWF), fibrin D-dimer, N-terminal pro-brain natriuretic peptide (NT-ProBNP), and cardiac Troponin T (cTnT)

For peer review only

References

1. Pocock SJ, Ashby D, Shaper AG*, et al.* Diurnal variations in serum biochemical and haematological measurements. *Journal of clinical pathology*. 1989; 42: 172-9.
2. Emberson JR, Whincup PH, Walker M*, et al.* Biochemical measures in a population-based study: effect of fasting duration and time of day. *Annals of clinical biochemistry*. 2002; 39: 493-501.
3. Elliott WJ. Circadian variation in blood pressure: Implications for the elderly patient. *American Journal of Hypertension*. 1999; 12: 43S-9S.
4. IL6R Genetics Consortium and Emerging Risk Factors Collaboration. Interleukin-6 receptor pathways in coronary heart disease: a collaborative meta-analysis of 82 studies. *The Lancet*. 2012; 379: 1205-13.
5. Wannamethee SG, Shaper AG, Papacosta O*, et al.* Lung function and airway obstruction: associations with circulating markers of cardiac function and incident heart failure in older men-the British Regional Heart Study. *Thorax*. 2016; 71: 526-34.
6. Rudnicka AR, Rumley A, Lowe GD*, et al.* Diurnal, seasonal, and blood-processing patterns in levels of circulating fibrinogen, fibrin D-dimer, C-reactive protein, tissue plasminogen activator, and von Willebrand factor in a 45-year-old population. *Circulation*. 2007; 115: 996-1003.
7. Natriuretic Peptides Studies Collaboration. Natriuretic peptides and integrated risk assessment for cardiovascular disease: an individual-participant-data meta-analysis. *The lancet Diabetes & endocrinology*. 2016; 4: 840-9.
8. Shaper AG, Pocock SJ, Walker M*, et al.* British Regional Heart Study: cardiovascular risk factors in middle-aged men in 24 towns. *BMJ*. 1981; 283: 179-86.
9. Pocock SJ, Shaper AG, Cook DG*, et al.* British Regional Heart Study: geographic variations in cardiovascular mortality, and the role of water quality. *British Medical Journal*. 1980; 280: 1243-9.
10. Declaration of Helsinki - Ethical Principles for Medical Research Involving Human Subjects. *WMA Declaration of Helsinki*. WMA Declaration of HelsinkiOctober 2013.
11. Wannamethee G and Shaper AG. Physical activity and stroke in British middle aged men. *BMJ : British Medical Journal*. 1992; 304: 597-601.
12. Jefferis BJ, Sartini C, Ash S*, et al.* Validity of questionnaire-based assessment of sedentary behaviour and physical activity in a population-based cohort of older men; comparisons with objectively measured physical activity data. *Int J Behav Nutr Phys Act*. 2016; 13: 14.
13. Sartini C, Barry SJ, Wannamethee SG*, et al.* Effect of cold spells and their modifiers on cardiovascular disease events: Evidence from two prospective studies. *Int J Cardiol*. 2016; 218: 275-83.
14. Stolwijk AM, Straatman H and Zielhuis GA. Studying seasonality by using sine and cosine functions in regression analysis. *Journal of Epidemiology and Community Health*. 1999; 53: 235-8.
15. Wannamethee SG, Whincup PH, Lennon L*, et al.* Associations Between Fibrin D-Dimer, Markers of Inflammation, Incident Self-Reported Mobility Limitation, and All-Cause Mortality in Older Men. *Journal of the American Geriatrics Society*. 2014; 62: 2357-62.
16. Nilsonne G, Lekander M, Åkerstedt T*, et al.* Diurnal variation of circulating interleukin-6 in humans: a meta-analysis. *bioRxiv*. 2016.

For peer review only

17. Gudewill S, Pollmacher T, Vedder H, *et al.* Nocturnal plasma levels of cytokines in healthy men. *European archives of psychiatry and clinical neuroscience*. 1992; 242: 53-6.
18. Bauer J, Hohagen F, Ebert T, *et al.* Interleukin-6 serum levels in healthy persons correspond to the sleep-wake cycle. *The Clinical investigator*. 1994; 72: 315-.
19. Gimeno D, Kivimaki M, Brunner EJ, *et al.* Associations of C-reactive protein and interleukin-6 with cognitive symptoms of depression: 12-year follow-up of the Whitehall II study. *Psychological medicine*. 2009; 39: 413-23.
20. Sartini C, Wannamethee SG, Iliffe S, *et al.* Diurnal patterns of objectively measured physical activity and sedentary behaviour in older men. *BMC Public Health*. 2015; 15: 609.
21. Goetze JP, Jorgensen HL, Sennels HP, *et al.* Diurnal plasma concentrations of natriuretic propeptides in healthy young males. *Clinical chemistry*. 2012; 58: 789-92.
22. Eliasson M, Evrin PE, Lundblad D, *et al.* Influence of gender, age and sampling time on plasma fibrinolytic variables and fibrinogen. *Fibrinolysis*. 1993; 7: 316-23.
23. Meier-Ewert HK, Ridker PM, Rifai N, *et al.* Absence of diurnal variation of C-reactive protein concentrations in healthy human subjects. *Clinical chemistry*. 2001; 47: 426-30.
24. Jafri SM, VanRollins M, Ozawa T, *et al.* Circadian variation in platelet function in healthy volunteers. *Am J Cardiol*. 1992; 69: 951-4.
25. Bridges AB, McLaren M, Scott NA, *et al.* Circadian variation of tissue plasminogen activator and its inhibitor, von Willebrand factor antigen, and prostacyclin stimulating factor in men with ischaemic heart disease. *British Heart Journal*. 1993; 69: 121-4.
26. Klinkenberg LJ, van Dijk JW, Tan FE, *et al.* Circulating cardiac troponin T exhibits a diurnal rhythm. *Journal of the American College of Cardiology*. 2014; 63: 1788-95.
27. Bremner WF, Sothern RB, Kanabrocki EL, *et al.* Relation between circadian patterns in levels of circulating lipoprotein(a), fibrinogen, platelets, and related lipid variables in men. *American heart journal*. 2000; 139: 164-73.
28. Kristal-Boneh E, Harari G and Green MS. Seasonal Change in 24-Hour Blood Pressure and Heart Rate Is Greater Among Smokers Than Nonsmokers. *Hypertension*. 1997; 30: 436-41.
29. Vgontzas AN, Zoumakis M, Bixler EO, *et al.* Impaired nighttime sleep in healthy old versus young adults is associated with elevated plasma interleukin-6 and cortisol levels: physiologic and therapeutic implications. *J Clin Endocrinol Metab*. 2003; 88: 2087-95.
30. Elliott WJ. Circadian variation in the timing of stroke onset: a meta-analysis. *Stroke; a journal of cerebral circulation*. 1998; 29: 992-6.
31. Carson PA, O'Connor CM, Miller AB, *et al.* Circadian rhythm and sudden death in heart failureResults from prospective randomized amlodipine survival trial. *Journal of the American College of Cardiology*. 2000; 36: 541-6.

For peer review only

Figure 1 – Unadjsted geometric means (95% CI) by time of the day [1] for C-Reactive Protein (CRP), Interleukin-6 (IL-6), Fibrinogen, tissue plasminogen activator (t-PA) , von Willebrand factor (vWF), fibrin D-dimer, N-terminal pro-brain natriuretic peptide (NT-ProBNP), and cardiac Troponin T (cTnT) measured on one occasion in BRHS men aged 60-79 during the years 1998-2000.

[1] Total number of men examined per hour was 33(0.7%) at 08:00-08:59, 363(8.5%) at 8:00-9:59h, 699(16%) at 10:00-10:59h, 771(18%) at 11:00-11:59h, 591(14%) at 12:00-12:59h, 99(2%) at 13:00-13:59h, 306(7%) at 14:00-14:59), 560(13%) at 15:00-15:59h, 566(13%) at 16:00-16:59, 260(6%) at 17:00-17:59, and 3(<0.1%) at 18:00-18:59

For peer review only

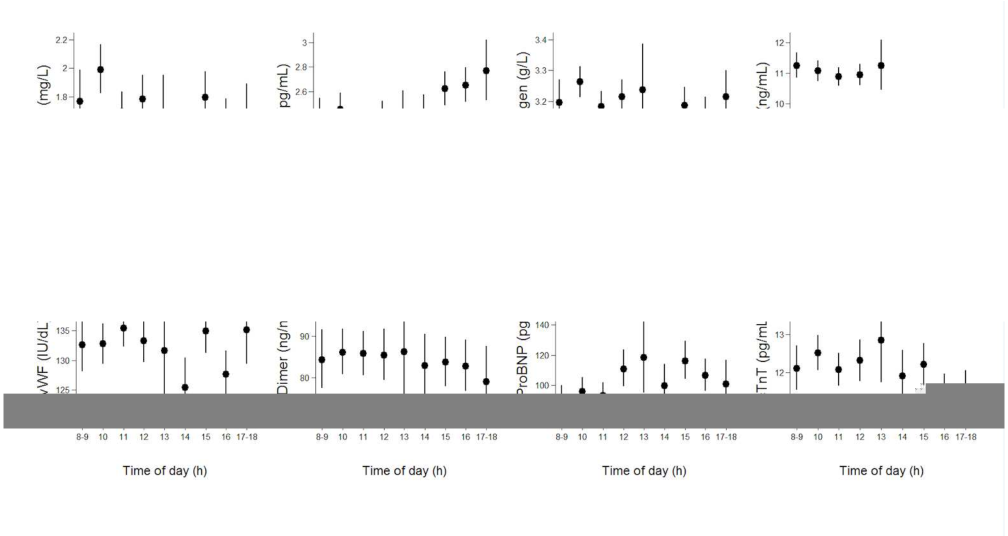

Figure 1 – Unadjsted geometric means (95% CI) by time of the day

186x99mm (300 x 300 DPI)

review only

STROBE Statement—Checklist of items that should be included in reports of ***cross-sectional studies***

	Item No	Recommendation	
**Title and abstract**	1	(*a*) Indicate the study's design with a commonly used term in the title or the abstract	Abstract
		(*b*) Provide in the abstract an informative and balanced summary of what was done and what was found	Abstract
**Introduction**			
Background/rationale	2	Explain the scientific background and rationale for the investigation being reported	Introduction, lines 54-62
Objectives	3	State specific objectives, including any prespecified hypotheses	Introduction, lines 54-56 and 60-62
**Methods**			
Study design	4	Present key elements of study design early in the paper	Methods, line 66-72
Setting	5	Describe the setting, locations, and relevant dates, including periods of recruitment, exposure, follow-up, and data collection	Methods, lines 75-84
Participants	6	(*a*) Give the eligibility criteria, and the sources and methods of selection of participants	Methods, lines 66-72 snd 75-84
Variables	7	Clearly define all outcomes, exposures, predictors, potential confounders, and effect modifiers. Give diagnostic criteria, if applicable	Methods , lines 97-123
Data sources/ measurement	8*	For each variable of interest, give sources of data and details of methods of assessment (measurement). Describe comparability of assessment methods if there is more than one group	Methods , lines 75-123
Bias	9	Describe any efforts to address potential sources of bias	
Study size	10	Explain how the study size was arrived at 4252	Results, lines 75-78
Quantitative variables	11	Explain how quantitative variables were handled in the analyses. If applicable, describe which groupings were chosen and why	Methods , lines 91-114
Statistical methods	12	(*a*) Describe all statistical methods, including those used to control for confounding	Methods , lines 125-159
		(*b*) Describe any methods used to examine subgroups and interactions	Methods , lines 152 and 156-158
		(*c*) Explain how missing data were addressed	
		(*d*) If applicable, describe analytical methods taking account of sampling strategy	NA
		(*e*) Describe any sensitivity analyses	Methods , lines 151-159
**Results**			
Participants	13*	(a) Report numbers of individuals at each stage of study—eg numbers potentially eligible, examined for eligibility, confirmed eligible, included in the study, completing follow-up, and analysed	Methods, , lines 66-68 and 91-95
		(b) Give reasons for non-participation at each stage	Methods, lines 66-76
		(c) Consider use of a flow diagram	Not necessary for this study, see Methods, lines 66-68 and 91-95

For peer review only - http://bmjopen.bmj.com/site/about/guidelines.xhtml

Descriptive data	14*	(a) Give characteristics of study participants (eg demographic, clinical, social) and information on exposures and potential confounders	Table 1
		(b) Indicate number of participants with missing data for each variable of interest	Table 1
Outcome data	15*	Report numbers of outcome events or summary measures	Table 1, Figure 1
Main results	16	(*a*) Give unadjusted estimates and, if applicable, confounder-adjusted estimates and their precision (eg, 95% confidence interval). Make clear which confounders were adjusted for and why they were included	Table 2
		(*b*) Report category boundaries when continuous variables were categorized	NA
		(*c*) If relevant, consider translating estimates of relative risk into absolute risk for a meaningful time period	NA
Other analyses	17	Report other analyses done—eg analyses of subgroups and interactions, and sensitivity analyses	Results, lines 187-195
**Discussion**			
Key results	18	Summarise key results with reference to study objectives	Results, lines 170-181 Discussion, lines 224-235
Limitations	19	Discuss limitations of the study, taking into account sources of potential bias or imprecision. Discuss both direction and magnitude of any potential bias	Discussion, lines lines 272-284
Interpretation	20	Give a cautious overall interpretation of results considering objectives, limitations, multiplicity of analyses, results from similar studies, and other relevant evidence	Discussion, lines 230-234 and 272 -284
Generalisability	21	Discuss the generalisability (external validity) of the study results	Discussion, lines 287-289
**Other information**			
Funding	22	Give the source of funding and the role of the funders for the present study and, if applicable, for the original study on which the present article is based	Acknowledgements, lines 287-302

*Give information separately for exposed and unexposed groups.

Note: An Explanation and Elaboration article discusses each checklist item and gives methodological background and published examples of transparent reporting. The STROBE checklist is best used in conjunction with this article (freely available on the Web sites of PLoS Medicine at http://www.plosmedicine.org/, Annals of Internal Medicine at http://www.annals.org/, and Epidemiology at http://www.epidem.com/). Information on the STROBE Initiative is available at www.strobe-statement.org.

For peer review only

For peer review only - http://bmjopen.bmj.com/site/about/guidelines.xhtml
